# A Design-by-Analogy Method for Integrating Chinese Paper-Cutting Art into Product Innovation: Understanding Attractiveness Associations via Expert Evaluations and Eye Tracking

**DOI:** 10.3390/biomimetics11080589

**Published:** 2026-08-18

**Authors:** Wenzhi Zhou, Tiantian Li

**Affiliations:** 1 Department of Product Design, Xiamen Academy of Arts and Design, Fuzhou University, Xiamen 361024, China; zhouwenzhi@fzu.edu.cn; 2 School of Industrial Design, Hubei Institute of Fine Arts, Wuhan 430205, China; 3 Academy of Arts & Design, Tsinghua University, Beijing 100085, China

**Keywords:** design-by-analogy, SCAMPER, paper-cutting, product innovation, communication model

## Abstract

Integrating traditional cultural heritage, such as Chinese paper-cutting, into modern product design is a critical challenge. To address the lack of a systematic method, this study proposes the PPI-SCAMPER method: a structured Design-by-Analogy (DbA) method that integrates knowledge bases (a Paper-Cutting Element Category and a Flat Process Category) with SCAMPER heuristics. Subsequently, an empirical study involving 18 experts and 146 users was conducted to evaluate the method’s outcomes. Multimodal data—including expert ratings of product attributes, user ratings of attractiveness, and eye-tracking data—were collected, and hierarchical regression and Random Forest models were employed to analyze the underlying mechanisms of attractiveness. The results indicate that products designed via PPI-SCAMPER achieved significantly higher ratings for user attractiveness, novelty, and culture attribute. Eye-tracking data revealed that the improved products elicited greater overall visual exploration. The exploratory analysis of associative patterns showed that product attributes constituted the primary basis for attractiveness judgments, with eye-tracking data providing significant yet limited incremental explanatory power. This study not only constructs an innovative design pathway for integrating paper-cutting culture from a DbA perspective but also provides a comprehensive framework for evaluating culturally inspired innovations through multi-dimensional empirical data.

## 1. Introduction

Chinese paper-cutting, as an ancient folk art originating in the 6th century A.D. [1], showcases its unique charm in the context of globalization with its distinctive visual language and profound cultural heritage. How to effectively integrate it into modern product design to achieve the revitalization and inheritance of traditional cultural heritage has become an important topic in the design field [2]. Widespread practices have been conducted; for example, researchers have used shape grammar theory to analyze the modern transformation of Fuyang paper-cutting [3], explored AI-assisted intelligent generation of Dong paper-cut patterns [4], or developed serious games to protect and promote the art form [5]. These explorations fully demonstrate the great potential and cultural added value of paper-cutting art in contemporary design. However, how to systematically integrate these rich traditional cultural elements into modern product development remains a challenge to be addressed.

To address this challenge, this study turns its focus to Analogical Design. Design-by-Analogy (DbA) is an effective tool for providing inspiration for innovative design [6,7,8,9]. The core of DbA lies in migrating and applying the solutions or knowledge structures of a “source domain” (e.g., paper-cutting art) to solve a problem in a “target domain” (e.g., modern products) [10]. This migration can be based on semantic (conceptual) features or visual (structural) representations [11]. It can be considered that integrating paper-cutting art into product development is, in essence, a typical analogical practice. It not only involves the translation of semantic features but also the reconstruction of visual representations (e.g., natural elements such as flowers, birds, fish, and insects, as well as abstract structures like symmetry and repetition), making it naturally suitable for the DbA framework. However, how to provide an operable method and systematic support for it remains a key issue. Research has confirmed that design based on cross-domain analogies may yield more creative results, which further raises the questions of how to initiate such analogical designs and what kind of help such a method can provide for the design process [12]. In recent years, the increasingly rich design databases and artificial intelligence (AI) technologies have brought new opportunities for developing data-driven methods and tools to support DbA. Researchers have begun to explore how to build more intelligent analogical design tools by addressing aspects such as analogical encoding, retrieval, mapping, and evaluation [13]. These advancements inspire us to consider whether classic DbA methods such as SCAMPER can be further improved [14,15,16] to better serve the complex task of cultural innovation design based on paper-cutting art. This constitutes the first core motivation of this study.

However, the effectiveness of an innovative design method cannot solely rest on the logical self-consistency of its construction; it also needs to be evaluated through empirical testing of its outputs to see if the designed products are genuinely recognized by different audiences. The core challenge of this evaluation process lies in verifying the consistency between “designer’s intent” and “user’s interpretation”. As revealed by Crilly et al. in the Design as Communication Model, designers transmit information (intent) to users through the product (medium), and users decode this information (interpretation) through perception and experience [17,18]. Its essence originates from the perspective of information theory, namely, Shannon’s model of information transmission [19,20]. This communication model not only focuses on the information transmission between the product and the audience but also implies the potential cognitive gap that may arise among different audiences (such as experts and users) due to differences in their background knowledge and decoding abilities [21,22]. Therefore, verifying whether products designed with the new method can more effectively convey a positive perception to different audiences becomes the second core motivation of this study.

Cultural products, due to the specific added value they carry, aim to create a positive user experience by evoking users’ perceptions and emotions [23,24,25]. The formation mechanism of their user attractiveness is often more complex than that of general products, requiring a more in-depth research framework. In recent years, with the development of physiological measurement technologies, researchers have begun to predict product attractiveness by integrating multi-dimensional data through empirical methods [26,27,28,29]. Biometric measures can capture people’s unconscious and involuntary behaviors [30,31], which is very useful for eliciting people’s hidden thoughts and emotional responses [32]. These research methods can be broadly categorized into two research paradigms: “computational aesthetics” and “aesthetic computing” [33,34]. Computational aesthetics focuses on the morphological features of the product itself, analyzing objective attributes such as form and complexity through algorithms. Aesthetic computing, on the other hand, focuses on the user’s side, measuring subjective experiences and physiological responses during the perception process through experimental methods such as eye tracking [35,36]. Previous studies have often focused on only one of these aspects. This study argues that a complete investigation of the influence mechanism must combine both, by jointly considering the product attributes representing “designer’s intent” (expert evaluations of form, belonging to the realm of computational aesthetics) and the visual behaviors representing “user’s interpretation” (user eye-tracking data, belonging to the realm of aesthetic computing). Therefore, exploring the correlation patterns among product attributes, eye-tracking metrics, and users’ overall attraction to the product constitutes the third and most integrative core motivation of this study.

Based on the above rationale, this study addresses the following three research questions:

**RQ1:**  *How can an innovative design method be developed based on Design-by-Analogy (DbA) to integrate traditional Chinese paper-cutting art?*

**RQ2:**  *Do the improved products designed using this method outperform the original products in terms of expert evaluations, user visual behaviors, and subjective attractiveness?*

**RQ3:**  *What is the relationship among product attributes (as assessed by experts), users’ eye-tracking behavior (objectively measured), and product attractiveness (as rated by users)?*

To systematically address these questions, the remainder of this paper is structured as follows: Section 2 reviews the relevant literature on analogical design and innovative product evaluation and elaborates on the proposed PPI-SCAMPER design method. Section 3 outlines the mixed-methods approach adopted in this study, including expert questionnaires, user eye-tracking experiments, and subjective evaluation questionnaires, as well as the data analysis strategies. Section 4 presents the detailed results of the data analysis. Finally, Section 5 provides an in-depth discussion of the results centered on the three research questions, and Section 6 concludes the paper by summarizing the study’s contributions, limitations, and directions for future research. This study aims to contribute a practical, DbA-informed method for integrating cultural elements into modern product innovation and to offer preliminary empirical evidence for examining how such innovative products are perceived and evaluated.

## 2. Related Works

### 2.1. Analogical Design

Analogical reasoning is widely recognized as a pivotal cognitive strategy in design, particularly for innovative design endeavors. Research in this field emphasizes that a profound exploration of creativity must be situated at the intersection of cognitive science, social psychology, and engineering design [6]. At its core, analogical reasoning is a process of knowledge transfer, wherein knowledge from a familiar source domain is retrieved and applied to solve a problem in a current target domain. This analogical transfer can occur at various levels: within a single problem (within-problem), across different problems within the same domain (within-domain), or even across disparate domains (cross-domain) [37]. The prevailing view in the literature is that successful analogy-making hinges on the designer’s mental models, which guide both the selection of what to match and the evaluation of how well it matches [8,38,39,40,41]. Concurrently, linguistics and semantic transfer, whether explicit or implicit, form a foundational basis for analogical reasoning [10].

In the practice of product design, analogical design manifests in several primary forms: Form Analogy (e.g., Biomimicry), Functional Analogy, Relational/Structural Analogy [42,43], and Symbolic/Cultural Analogy. Furthermore, analogical design is intimately linked with embodied cognition. Van et al. [44] developed a comprehensive framework for understanding the relationship between embodiment and the design experience, identifying four types of embodiment: (1) Anthropomorphism, Familiarity, and Literal Resemblances; (2) Relational Properties: Image Schemas and Symbolic Meaning; (3) Meaningful Sensorial Experiences; and (4) Embodiment in Product Movement and Action. The innovative product development based on Chinese paper-cutting is precisely such a comprehensive analogical design practice: it transcends mere borrowing of form, relying more deeply on analogies of its symbolic/cultural connotations and the stylistic translation of its visual language.

The degree of cross-domain transfer, known as “analogical distance”—the conceptual gap between the source and target domains—is a critical factor influencing design novelty [6]. Christensen’s research revealed that creativity tends to be constrained when analogies are made within the same domain [6]. Conversely, far-field analogies show a positive correlation with the originality of the design [45]. However, the analogical distance is not a simple case of “the farther, the better”; excessively remote stimuli can adversely affect the design process [7]. In essence, the appropriate type of analogy should be employed at different stages of the design process.

To systematically help designers engage in analogical thinking, researchers have developed various methods for systematically exploring the possibilities of analogy, such as Synectics [46] and WordTree [47]. A typical example is Biologically inspired design, which employs a composite analogy approach to generate new design concepts by integrating the results of multiple cross-domain analogies. This process typically involves four iterative steps: (1) presentation of the problem, (2) elaboration of the problem space, (3) retrieval of the analogue, and (4) application of the analogous solution [48]. Similarly, SCAMPER, as a semi-directed ideation method, has also been categorized as a Design-by-Analogy (DbA) method [14]. It utilizes seven operator categories (Substitute, Combine, etc.) to structure, expand, and extend brainstorming [49], with its operational logic being, in essence, a rule-guided form of analogical thinking. Studies indicate that compared to other DbA methods like WordTree, SCAMPER is more effective at enhancing novelty, though it is slightly less capable of mitigating design fixation [50]. Consequently, researchers have begun to explore combining SCAMPER with other design methods to leverage their respective strengths. Such integrative approaches have shown positive results; for instance, Hussain combined SCAMPER with analogies of animal form and function, leading to significantly higher creativity scores among student participants [51]. Advances in analogical reasoning for ideation also include the development of analogical search methods and search engines to identify potential analogies from digital resources, databases, and repositories [15,16].

### 2.2. PPI-SCAMPER: Developing an Innovative Design Method for Integrating Paper-Cutting Art

**Figure 1:**
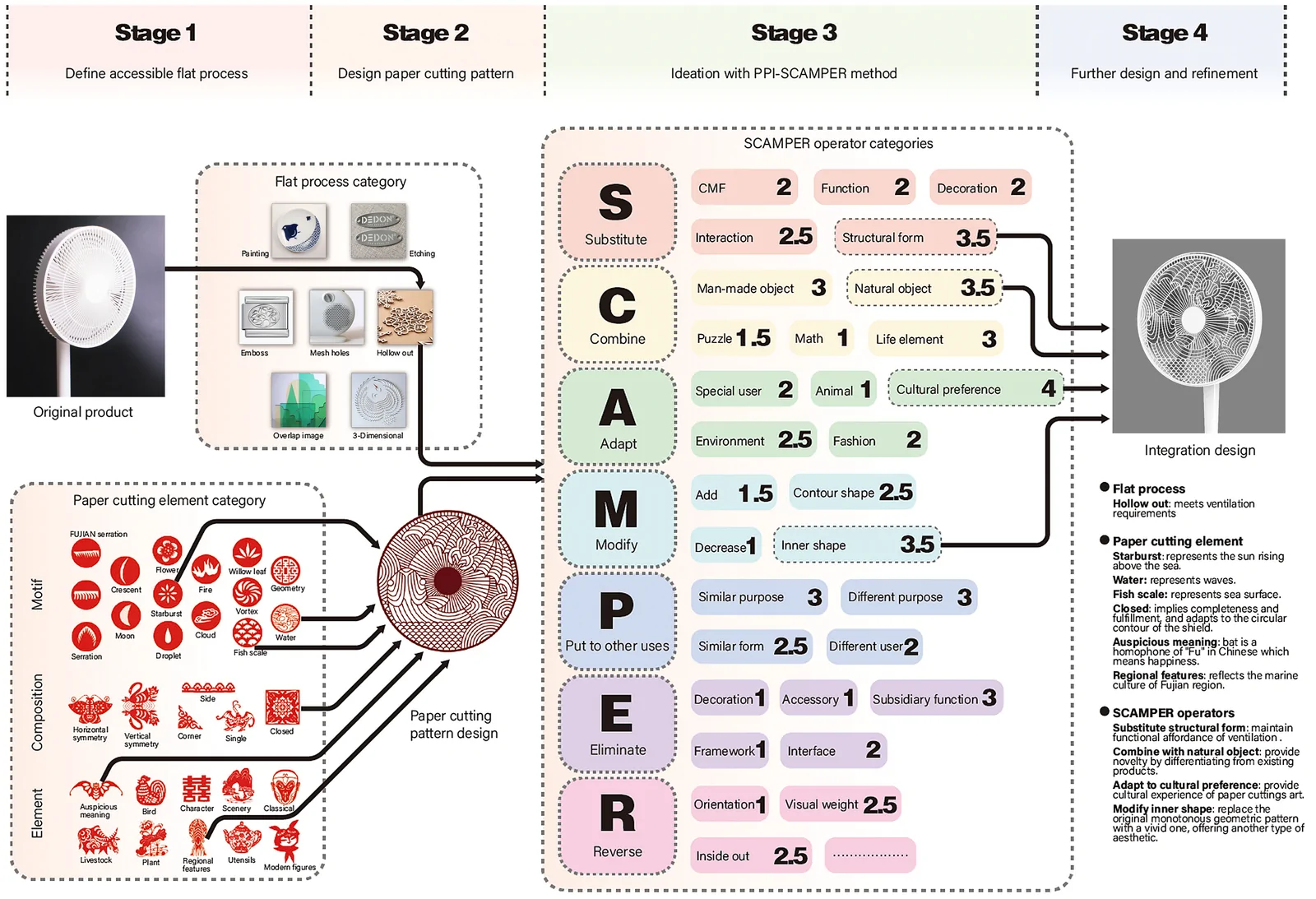
The design process flowchart of PPI-SCAMPER. The diagram illustrates the four-stage process of integrating a paper-cutting pattern into the fan guard design, using the fan as a case study.

While SCAMPER is a widely applied DbA method, its practical application faces three primary challenges. First, as a heuristic ideation tool [52,53], its strength lies in breaking cognitive fixation. However, the lack of explicit inputs and boundaries can easily lead to divergent thinking that strays from the core objective. Furthermore, research indicates that it is more inclined to build upon existing ideas rather than initiating new ones [54], thus necessitating the provision of materials closely related to the design theme, such as the integration of paper-cutting and products. Second, the sheer volume of ideas it generates makes it difficult to rapidly identify and screen for valuable concepts. Third, its application is often incomplete; an analysis of 39 relevant papers revealed that 59% of designers used only three or fewer of the seven operators [55], indicating that the method’s tooling and procedural aspects still need improvement. To systematically address these issues, we propose an integrated design method: PPI-SCAMPER (Paper-cutting and Product Integration-SCAMPER). Its core idea is to use the product’s achievable craft features (Flat Process) and the visual elements of paper-cutting (Paper-Cutting Element) as two independent inputs. These are then structurally combined and explored through the seven SCAMPER operators to systematically generate a large number of integrated concepts, thereby enhancing the creativity of the solutions [56] and guiding designers in their evaluation and selection. The method comprises four distinct stages as follows (Figure 1, using a fan as an example):

• **Stage 1: Define Accessible Flat Process.** This stage aims to identify the craft features on the product itself that can accommodate flat patterns. We established a Flat Process Category, which systematically includes common craft options. As shown in Figure 1, this category primarily consists of seven processes: Painting, Etching, Emboss, Mesh holes, Hollow out, Overlap image, and 3-Dimensional shaping. The significance of these processes varies: some (such as Painting) mainly provide purely visual decoration, contributing to the aesthetic and cultural experience, while others (such as Mesh holes for ventilation or Etching to increase friction) can, beyond decoration, be deeply integrated with product functions, bringing additional utility and novelty to the design.
• **Stage 2: Design Paper-Cutting Pattern.** The core of this stage is to create or select appropriate paper-cutting patterns based on the product’s form and functional requirements. To assist designers in this creative process, we constructed a Paper-Cutting Element Category based on prior research. This category deconstructs the art of paper-cutting into three components: (i) Element, the basic semantic unit of paper-cutting, including auspicious meanings, characters, scenery, classical patterns, and regional features and utensils with local characteristics; (ii) Composition, the way elements are organized, which determines the overall structure of the pattern, such as horizontal symmetry, vertical symmetry, corner motifs, single compositions, and closed forms; and (iii) Motif, the specific visual forms that constitute the pattern, like starbursts, water waves, fish scales, flowers, and geometric shapes. Notably, this category emphasizes elements with Fujian regional characteristics (FUJIAN serration), facilitating the creation of works imbued with specific local culture. At this stage, designers must also consider two key dimensions: first, the constraints imposed by the product’s flat processes on the paper-cutting’s visual language (e.g., etching and embossing cannot show color, mesh holes make it difficult to form clear edges, and hollow-out designs require uniform and slender internal lines); second, the issue of adapting the form when extracting structural elements from the category to fit the product.
• **Stage 3: Ideation with the PPI-SCAMPER Method.** This is the core of the method. Designers take the outputs from the first two stages (one or more processes, one or more paper-cutting patterns) as inputs and apply the seven SCAMPER operator categories (Substitute, Combine, Adapt, Modify, Put to other uses, Eliminate, Reverse) to brainstorm and generate a multitude of integrated concepts. For example, as shown in the final design on the right of Figure 1, the Hollow Out process was selected and combined with a Starburst pattern representing “sunrise”. Simultaneously, a designer might use the Adapt operator to fit the fan’s circular contour and cultural preferences and the Modify operator to optimize the internal geometric form. This process yields many ideas, making a comprehensive evaluation after concept divergence essential.
• **Stage 4: Further Design and Refinement.** This stage is no different from a traditional design process. Based on the optimal concept solution selected in the third stage, designers carry out subsequent detailed design work, such as sketch ideation, rendering, and 3D modeling, until the final product design is complete.

**Figure 2:**
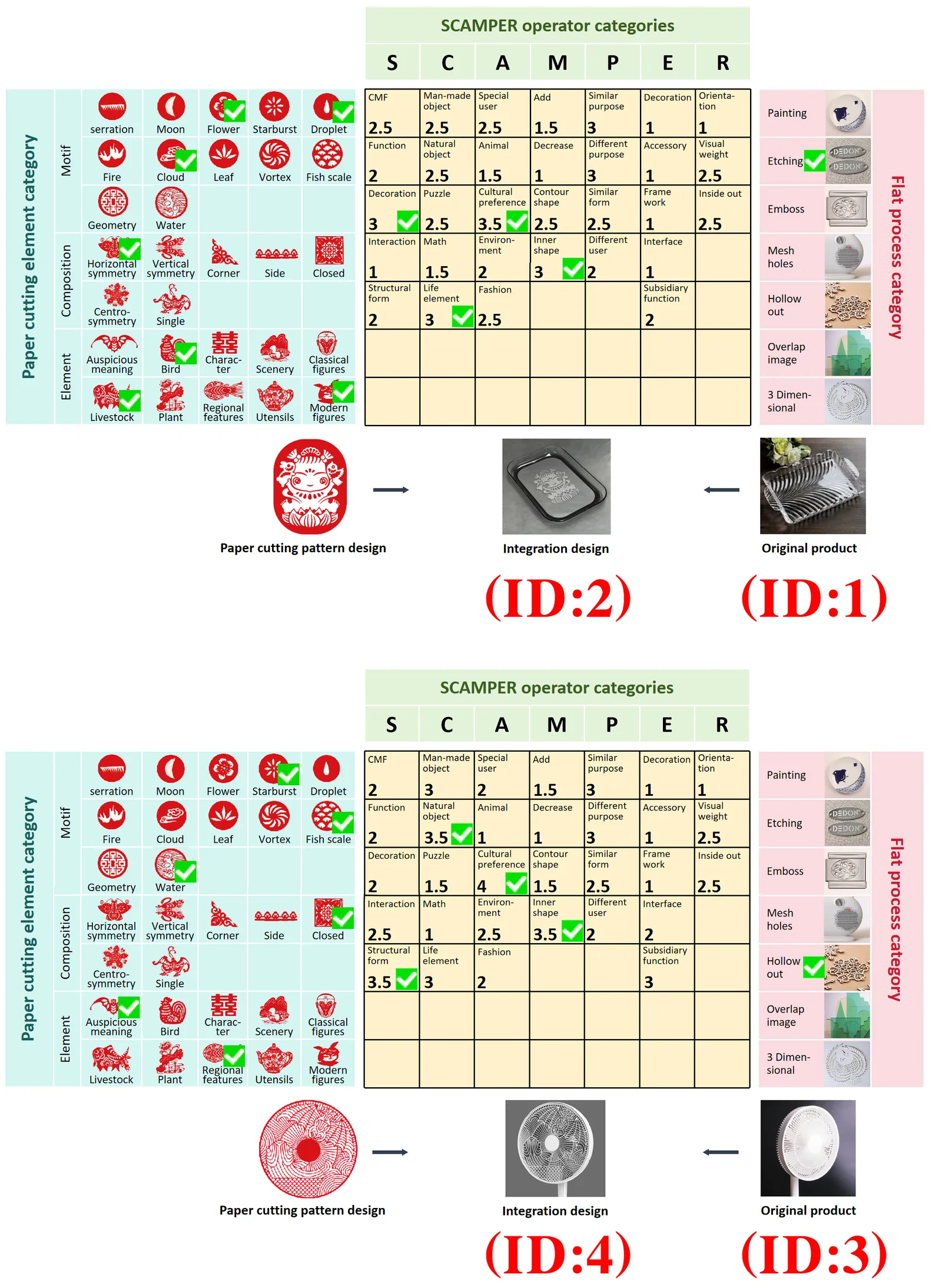
Illustrative application of the PPI-SCAMPER method for Chinese paper-cutting art product innovation. This figure showcases two design case studies: a tray (comparing ID:1 with ID:2) and a fan (comparing ID:3 with ID:4).

**Figure 3:**
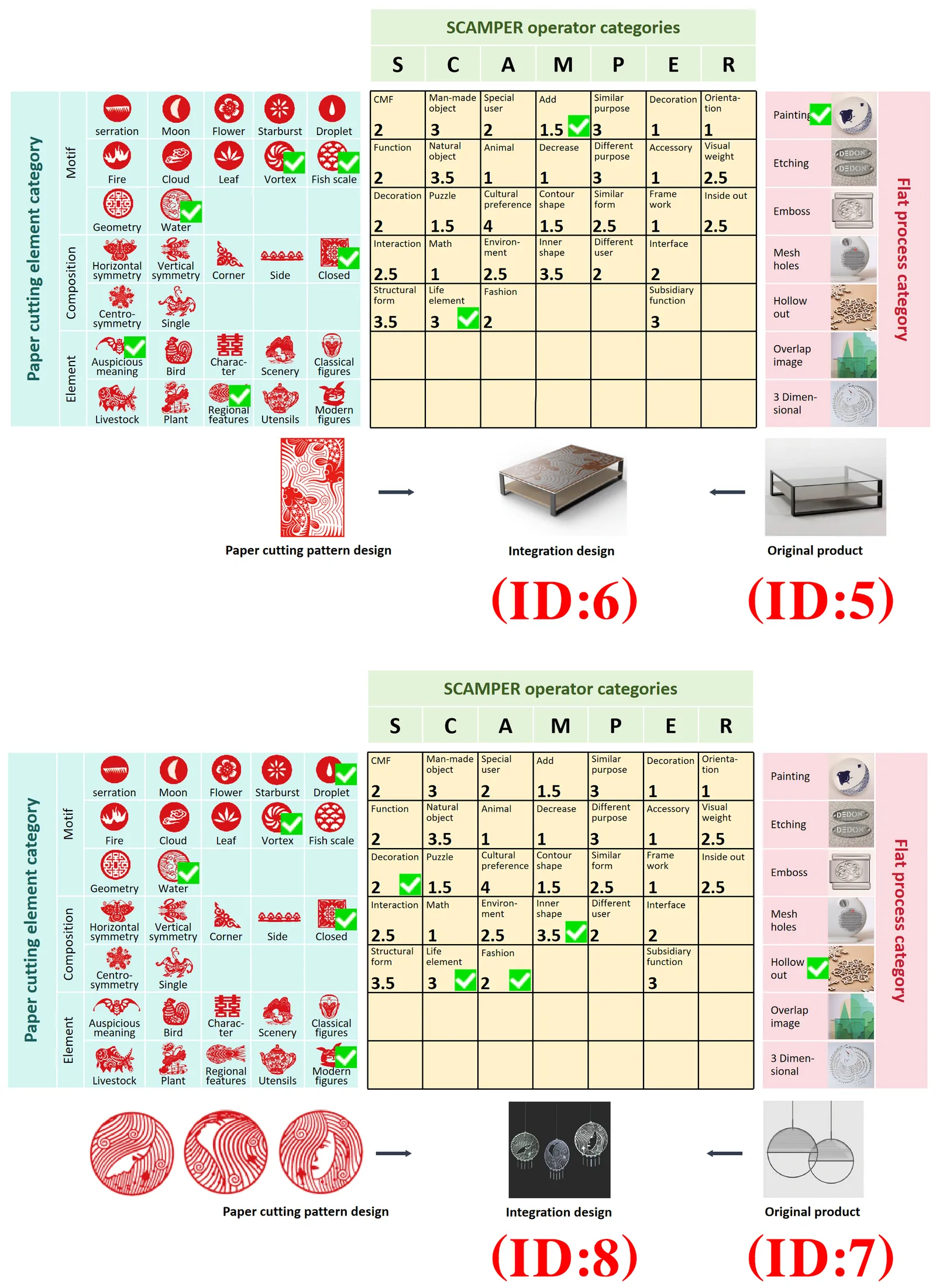
Illustrative application of the PPI-SCAMPER method for Chinese paper-cutting art product innovation. This figure showcases two design case studies: a side table (comparing ID:5 with ID:6) and a pendant set (comparing ID:7 with ID:8).

In addition to the fan, we have also completed three other integrated designs, including a glass tray, a coffee table, and a set of hanging decorations. All the images and design elements of the proposals are showed in Figure 2 and Figure 3, which will serve as materials for subsequent evaluation testing and analysis.

### 2.3. Evaluation Dimensions and Framework for Innovative Products

#### 2.3.1. Product Attribute Evaluation Dimensions: Aesthetic, Functional, Novelty, and Cultural

The evaluation of product attributes is rooted in their physical and perceptual characteristics. At a macro level, Hu et al. [57] proposed a five-element evaluation standard comprising the Degree of Innovation (DOI), Aesthetic Quality (AQ), Realization Possibility (RP), Functionality (FN), and Emotional Content (EC). From a value perspective, Blijlevens [58] and Hu et al. [59] have emphasized the importance of aesthetic pleasure and aesthetic indicators in product evaluation, while Christensen [60] explicitly identified functional value and aesthetic value as two high-level claims. In the realm of innovative design, Sarkar et al. [61] assess the creativity of products as a function of their novelty and usefulness. Furthermore, the intimate connection between culture and design has long been evident in the field of social anthropology [62]. In product design, the goal of cultural respect can be achieved by incorporating the historical and aesthetic values of users [63]. In summary, the assessment of product attributes typically encompasses the core dimensions detailed below (Table 1).

#### 2.3.2. User Visual Behavior Dimensions

Eye-tracking technology, by virtue of its objectivity and real-time capabilities, is increasingly applied in sensory and consumer science [74] and other fields [75,76,77]. It serves as an effective supplement to self-reports [78] and a predictor of product preference [79]. In product design and evaluation, this technology is frequently used to unveil patterns of visual attention and cognitive processing. For instance, Hu et al. [57] combined eye tracking with questionnaires and interviews to evaluate product designs, while Wang et al. [80] integrated visual sequence data from eye tracking with an improved LSTM model to enhance the emotional experience and satisfaction with new energy vehicle intelligent cockpits (NEV-ICs). Zuo et al. [81] examined the relationship between gaze consistency and aesthetic consistency when viewing abstract art. To capture the multifaceted nature of user interest in elements and the depth of information processing, researchers often employ a variety of eye-tracking metrics. Among them, Li and Guo (2016) selected five indicators—time to first fixation, first fixation duration, total fixation duration, visit count, and pupil diameter—to assess automotive styling aesthetics [82]. Wang et al. [83] utilized a set of seven core metrics, including time to first fixation, fixation before, first fixation duration, total fixation duration, fixation count, visit count, and total visit duration, to investigate their correlation mechanisms with aesthetic evaluation. Building upon this foundation, the present study selects seven key eye-tracking metrics to objectively measure user visual behavior (Table 2).

#### 2.3.3. User Subjective Attractiveness Evaluation Dimensions: Liking and Recommendation

The evaluation of subjective attractiveness relies on user self-reports. Affective design can stimulate psychological feelings, enhance emotional satisfaction, and increase purchase intention [84]. Within this context, liking, as a function of the products’ sensory characteristics [85], aims to ascertain consumer preferences [86]. It is important to note that emotional satisfaction is also related to users’ willingness to recommend [87,88]. Willingness to Recommend is a key indicator for assessing loyalty and word-of-mouth potential. Host and Knie-Andersen [89] defined it as the “willingness to engage in publicity without monetary incentives.” Reichheld (2003) [90] introduced the Net Promoter Score (NPS), which uses the core question “how likely is it that you would recommend to a friend or colleague,” and it has since become the gold standard for measuring satisfaction and loyalty [91]. Based on this, the present study selects “Liking” and “Recommendation” as the core evaluation indicators for subjective attractiveness (Table 3).

#### 2.3.4. Integrated Evaluation Framework

In the design domain, ensuring that the designer’s intent is accurately interpreted by the user and positively influences their product perception and preference is a central challenge [18,21,98,99,100,101]. An interest in the relationship between designers’ intentions and consumers’ interpretations has prompted many design theorists to view design as an instance of communication [18]. To deeply investigate the influence mechanism of product attributes and eye-tracking indicators on product attractiveness (RQ3), this study constructs a multi-dimensional analytical framework. This framework is based on the classic “intent–interpretation” theoretical model [17,18] and is combined with human factors empirical methods to avoid the potential lack of explanatory power that can arise from relying solely on physiological data [32]. It is specifically composed of the following parts:

(1) Product Side: Product attributes are the direct carriers of the designer’s intent. Their evaluation universally covers core dimensions such as aesthetic quality [58,59,60], functionality [60,61], novelty [61], and culture attribute [63] (see Table 1).
(2) Artifact: The corresponding products, generated through an analogy-based innovative design method, serve as the communication medium in the form of images [18,57].
(3) User Side (Interpretation Process): The user’s interpretation process involves complex cognitive processing. This study employs eye-tracking technology to objectively record users’ real-time visual behaviors, such as time to first fixation, total fixation duration, and fixation count [83] (see Table 2).
(4) User Side (Response): The user’s subjective opinion of the attractiveness of the product is a manifestation of their integrated perception and preference. It is measured through indicators such as liking [85,86] and willingness to recommend [90,91], reflecting the user’s emotional connection and behavioral intentions (see Table 3).
(5) Modeling Approach: To effectively analyze the relationships among product attributes, user visual attention, and subjective evaluation, an appropriate data modeling method must be chosen. Compared to Structural Equation Modeling (SEM), which is commonly used to test variable relationships [102,103,104,105,106], Hierarchical Multiple Regression analysis [107,108,109] can more directly clarify the individual and cumulative contributions of product attributes and eye-tracking indicators to the outcome. To address potential nonlinearities and higher-order interactions between variables, this should be supplemented with machine learning models (such as CART, RT, AdaBoost, and GBDT) for validation [110].

## 3. Methodology

### 3.1. Research Questions and Hypotheses

**RQ1:**  *How can an innovative design method be developed based on Design-by-Analogy (DbA) to integrate traditional Chinese paper-cutting art?*

**RQ2:**  *Do the improved products designed using this method outperform the original products in terms of expert evaluations, user visual behaviors, and subjective attractiveness?*

**RQ3:**  *What is the relationship among product attributes (as assessed by experts), users’ eye-tracking behavior (objectively measured), and product attractiveness (as rated by users)?*

**H3a.**  *Product attributes (aesthetic quality, functionality, novelty, and cultural perception) will significantly influence the overall product attractiveness perceived by users.*

**H3b.**  *Visual behavior patterns measured by eye tracking will contribute additional explanatory power for product attractiveness, above and beyond the effect of product attributes.*

**Figure 4:**
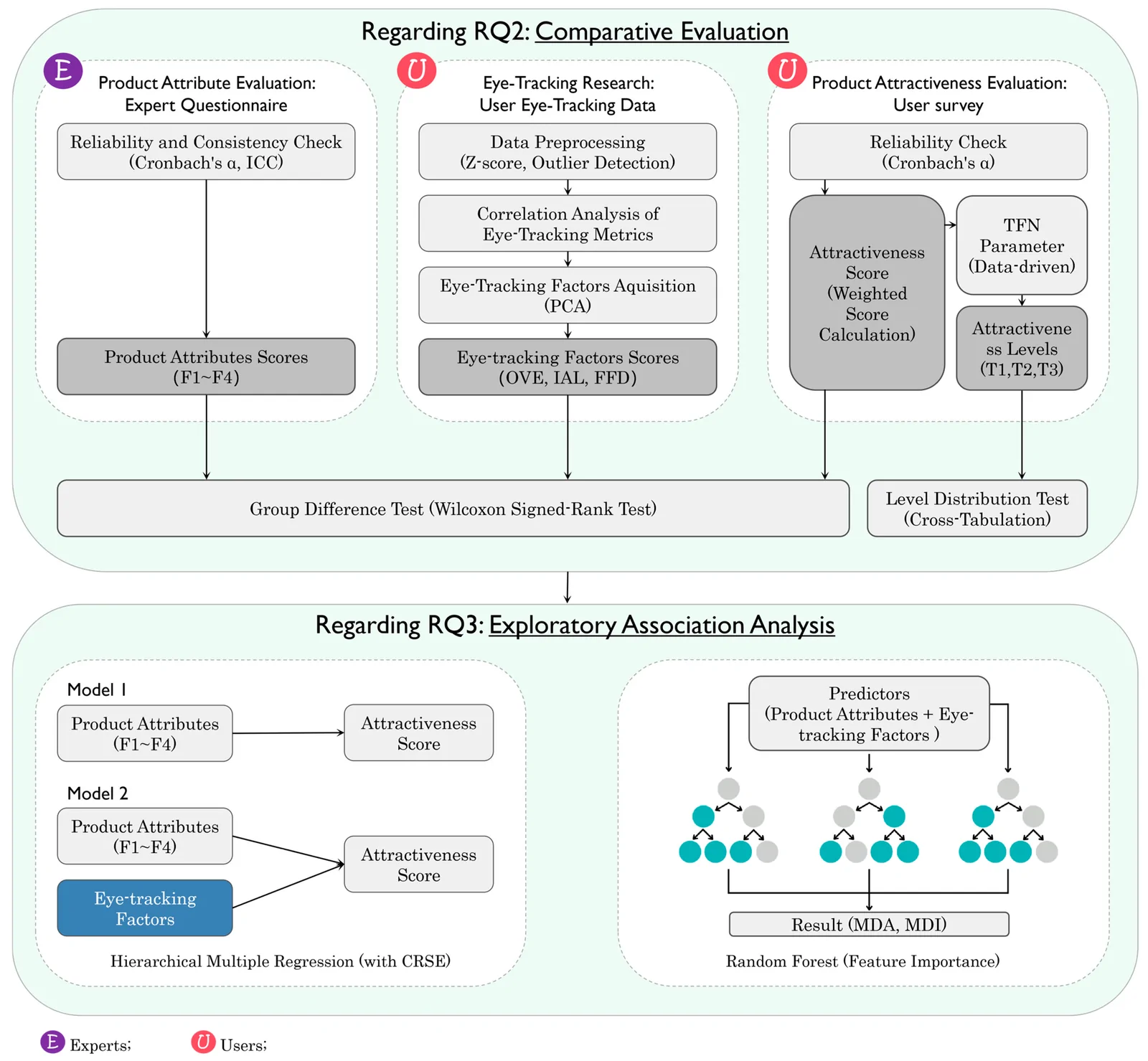
The overall research methodology framework.

This section primarily focuses on the evaluation of the innovative design’s effectiveness and the investigation of its underlying mechanisms (see Figure 4, addressing RQ2 and RQ3). The development process of the innovative design method, PPI-SCAMPER, has been detailed in Section 2. This process generated the product stimuli required for the present study (4 original products and 4 improved products).

### 3.2. Research Methods

#### 3.2.1. Expert Questionnaire: Product Attribute Evaluation

**Materials:** Eight products were selected, organized into four pairs. Each pair consisted of one product incorporating paper-cutting elements and one original product (see Section 2.2). To ensure the rigor of the eye-tracking experiment and perceptual evaluations, the study maintained consistency between the original and improved groups in terms of viewing angle, compositional proportions, and rendering quality, thereby avoiding the interference of extraneous variables.

**Participants:** The participants were comprised of 18 domain experts. Their demographic information is presented in Table 4.

**Procedure:** In the questionnaire, the 18 experts rated the four original and four improved products based on four key indicators: aesthetic quality, functionality, novelty, and culture attribute. A 5-point Likert scale was used for the evaluation, where the scores from 1 to 5 represented Strongly Dislike/Disagree, Dislike/Disagree, Neutral, Like/Agree, and Strongly Like/Agree, respectively.

**Data Analysis:** The analysis followed the steps below:

**Step (i)—Reliability Check:** Cronbach’s Alpha was calculated to assess the internal consistency of the scale. The Intraclass Correlation Coefficient (ICC) was computed to evaluate the degree of agreement among the 18 expert raters.

**Step (ii)—Descriptive Statistics:** The mean and standard deviation for each product on the four indicators were calculated to understand the basic distribution characteristics of the expert ratings.

#### 3.2.2. User Eye-Tracking Data: Visual Behavior Measurement and Factor Extraction

**Materials:** Eight products were selected, organized into four pairs, with each pair consisting of one product incorporating paper-cutting elements and one original product.

**Participants:** The participants were 146 students from Fuzhou University (Table 5). Prior to participating in the experiment, the participants were informed of the relevant factors related to the experiment, including the experimenter’s identity and affiliation, the purpose of the experiment, the experimental setup and format, the potential effects of the experiment on the participants, and the information the experimenter needed to collect from them. The participants took part in this experiment after being fully informed, giving their consent, and signing the informed consent form.

**Apparatus:** The eye tracker used in this experiment was the Tobii Pro X2-60, a non-invasive eye tracker. This desktop-mounted device has a sampling rate of 60 Hz, a gaze-positioning accuracy of 0.5°, and an operating distance of 40–90 cm. The stimuli were displayed on a 13-inch monitor, positioned approximately 65 cm from the participants’ eyes. The participant’s head was allowed to move within a 30 × 22 × 30 cm range. The device’s performance parameters were set to the default recommended settings.

**Procedure:** Before the formal experiment began, participants had to first undergo gaze calibration using the eye tracker and the screen. This experiment uses the five-point calibration method, in which participants must sequentially fixate on five dots that appear in the corners and center of the screen; during this process, the eye tracker determines the position and orientation of the participant’s eyes. As is standard practice, participants who were unable to complete eye tracking during calibration or whose effective sampling rate fell below 70% were excluded from the experiment. During the main experiment, stimuli were presented on the monitor, and participants were allowed minor head movements while viewing them. The instructions were as follows: “You will see a series of 4 groups of images one by one. You may view each image for as long as you like. To proceed to the next image, please press the spacebar.” Participants first read the instructions for 5 s. The viewing time for each image was self-paced, and they advanced to the next image by clicking the mouse until all four groups of images had been observed. Throughout this process, the eye tracker recorded the participants’ gaze paths and temporal data, allowing for the analysis of their interest in the image content.

**Figure 5:**
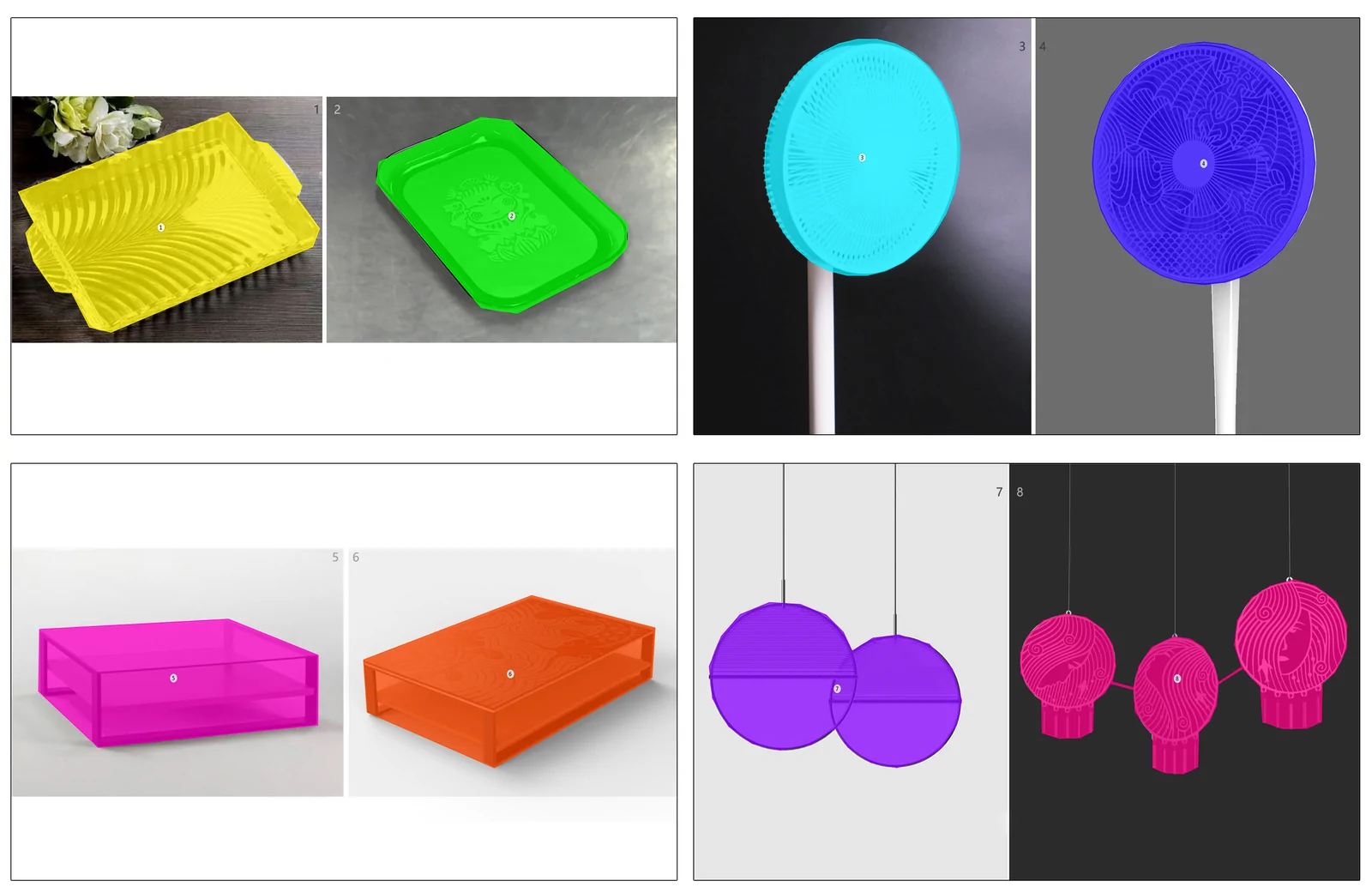
The eight product stimuli and their corresponding Area of Interest (AOI) definitions. In the eye-tracking data analysis, only gaze points falling within these predefined AOIs were included for calculation.

**Data Analysis:** The following steps were performed for data analysis:

**Step (i)—Define the AOI:** Define the AOI on the test material based on the edge contours of each product object; eye-tracking data within the AOI was collected. The four test images contain eight products, and the corresponding eight AOIs are shown in Figure 5.

**Step (ii)—Data Preprocessing:** After filtering out invalid records based on the aforementioned 70% valid sampling rate threshold, the eye-tracking data within the AOI underwent further preprocessing, including the detection and handling of obvious erroneous records and missing values. The seven eye-tracking metrics were standardized using Z-scores. Subsequently, to identify significant outliers, leverage values and Cook’s distances were calculated and jointly considered. The formula for leverage is as follows:

(1) $$h_{i}=\frac{1}{n}+\frac{{\left(x_{i}-\overset{-}{x}\right)}^{2}}{\sum_{j=1}^{n}{\left(x_{j}-\overset{-}{x}\right)}^{2}}$$

where $h_{i}$ is the leverage of the *i*th observation, *n* is the sample size, $x_{i}$ is the *i*-th observation, and $\overset{-}{x}$ is the mean of the independent variable.

The formula for Cook’s distance is:

(2) $$D_{i}=\frac{r_{i}^{2}}{p\cdot MSE}\cdot \frac{h_{i}}{{\left(1-h_{i}\right)}^{2}}$$

where $D_{i}$ is the Cook’s distance of the *i*th observation, $r_{i}$ is its standardized residual, *p* is the number of model parameters, and MSE is the mean squared error.

**Step (iii)—Correlation Analysis of Eye-Tracking Metrics:** Pearson correlation coefficients were calculated among the seven raw eye-tracking metrics to identify potential issues of multicollinearity.

**Step (iv)—Principal Component Analysis (PCA) of Eye-Tracking Metrics:** PCA was performed on the seven standardized eye-tracking metrics. The number of principal components to retain was determined using the standard criterion (Eigenvalue > 1), and an appropriate rotation method was applied to optimize the component structure.

#### 3.2.3. User Questionnaire: Subjective Attractiveness Score Acquisition and Level Classification

**Procedure:** After completing the eye-tracking experiment, the participants (see Table 5) were asked to fill out a product attractiveness questionnaire. In this questionnaire, participants evaluated each of the eight products in terms of Liking and Willingness to Recommend. A 5-point Likert scale was employed for the ratings, where the scores from 1 to 5 corresponded to Strongly Dislike/Disagree, Dislike/Disagree, Neutral, Like/Agree, and Strongly Like/Agree, respectively.

**Data Analysis:** The analysis was conducted in the following steps:

**Step (i)—Reliability Check:** Cronbach’s Alpha was calculated to assess the internal consistency and reliability of the questionnaire.

**Step (ii)—Weighted Score for Product Attractiveness:** Considering the importance of both Liking ($L_{i}$) and Willingness to Recommend ($R_{i}$) to product attractiveness [21,111], this study assigned equal weights (0.5 each) to both measures. Therefore, the product attractiveness score ($A_{i}$) was calculated as the weighted sum of these two ratings.

**Step (iii)—Determination of Triangular Fuzzy Number Parameters:** To scientifically handle the inherent ambiguity of subjective ratings, this study adopted a data-driven Triangular Fuzzy Numbers (TFNs) method for level classification. The core of this method is to use the distributional characteristics of the data itself (e.g., quartiles, skewness) to define the boundaries of the levels.

First, the upper and lower bounds of the rating interval were adjusted for skewness to accommodate the non-symmetrical distribution of the data. This adjustment was based on the data’s skewness and interquartile range (*IQR*):

(3) $$L_{bound}=\left\{\begin{array}{ll} min\left(X\right)-|Skew|\;\times \;IQR & ifSkew<0\\ min\left(X\right) & ifSkew\geq 0 \end{array}\right.$$

(4) $$U_{bound}=\left\{\begin{array}{ll} max\left(X\right)+|Skew|\;\times \;IQR & ifSkew>0\\ max\left(X\right) & ifSkew\leq 0 \end{array}\right.$$

Subsequently, based on this adjusted interval and the data’s quartiles, the TFN parameters (a, b, c) for the three attractiveness levels were defined as follows: Tier 3 (relatively disadvantaged group): $\left(L_{bound}\right.,P25,P50)$; Tier 2 (relatively moderate group): (*P*25, *P*50, *P*75); Tier 1 (relatively advantaged group): $\left(P50,P75,U_{bound}\right)$. It is important to note that tiers are relative classifications based on the data distribution; they reflect only a product’s relative ranking within the current sample pool rather than a value judgment based on an absolute scale (such as the midpoint on a Likert scale).

**Step (iv)—Attractiveness Level Classification:** The mean attractiveness score of each product (${\overset{¯}{A}}_{i}$) was applied to the TFNs to calculate the membership degrees of each product in the three relative tiers: Tier 1, Tier 2, and Tier 3. The final aesthetic evaluation level for each product was determined using the principle of maximum membership. The definition of the triangular membership function is:

(5) $$\mu_{i}\left(j\right)=\left\{\begin{array}{ll} 0, & if\;{\overset{-}{A}}_{i}\leq a_{j}\;or\;\geq c_{j}\\ \frac{{\overset{-}{A}}_{i}-a_{j}}{b_{j}-a_{j}}, & if\;a_{j}<{\overset{-}{A}}_{i}\leq b_{j}\\ \frac{c_{i}-{\overset{-}{A}}_{i}}{c_{j}-b_{j}}, & if\;b_{j}<{\overset{-}{A}}_{i}<c_{j} \end{array}\right.$$

where $\mu_{i}\left(j\right)$ represents the membership degree of the *i*th product to the *j*th evaluation level, and $a_{j}$, $b_{j}$, $c_{j}$ are the TFN parameters for the *j*th evaluation level, where $j\in \left\{1,2,3\right\}$, corresponding to Tier 1, Tier 2, and Tier 3, respectively.

#### 3.2.4. Addressing RQ2: Comparative Evaluation of Original vs. Improved Products

This section aims to systematically compare the overall performance of the original and improved product groups. Given that this study employed a within-subjects design, in which the same group of participants (N = 146) and experts (N = 18) evaluated all products, the following statistical methods were used to analyze these non-independent repeated-measure data:

**Step (i)—Paired Nonparametric Comparison:** The Wilcoxon Signed-Rank Test was used to test the paired differences between the original and improved products in terms of product attributes, eye-tracking factors, and subjective attractiveness scores. This method not only effectively controls for systematic errors caused by individual differences but also robustly handles non-normally distributed data, ensuring the reliability of the analysis results.

**Step (ii)—Attractiveness Tier Distribution Analysis:** Cross-tabulation analysis was used to visually present the differences in the percentage distribution of the original product group and the improved product group across the three relative tiers.

#### 3.2.5. Addressing RQ3: An Exploratory Analysis of the Correlation Patterns of Product Attractiveness

This section aims to conduct an exploratory analysis of the relationship among product attributes, eye-tracking behavior and attractiveness.

**Step (i)—Hierarchical Multiple Regression Analysis:** First, a baseline model (Model 1) was constructed with only the product attributes (F1–F4) as predictors to test hypothesis H3a—whether product attributes significantly predict the overall product attractiveness perceived by users. Subsequently, the eye-tracking factor scores were added as a second block of predictors to the baseline model to form the full model (Model 2). Given that this study employs a within-subjects design and the observed data are not independent of one another, traditional OLS regression may underestimate the standard errors. To address this issue, all regression coefficients reported in this study are corrected using cluster-robust standard errors (CRSEs), with the cluster variable being the participant ID (Subject). Model evaluation was conducted at two levels: firstly, by comparing changes in explanatory power (Δ*R*^2^) to determine whether the inclusion of eye-tracking indicators contributed to an additional amount of variance explained; secondly, by testing the significance of the coefficients for each predictor variable in the comprehensive model (Model 2) (based on *t*-values adjusted for CRSEs) to assess whether each variable possessed independent predictive power.

Step (ii)—Random Forest Regression (RFR): To complement the hierarchical regression model, this study employs the Random Forest Regression (RFR) model as a nonparametric exploratory tool to investigate potential nonlinear relationships between the predictor variables and product appeal. The Random Forest model incorporates both the four product attributes identified by experts (F1–F4) and the three user eye-tracking metrics as input features, with the user’s subjective attractiveness score serving as the target variable. The data were randomly split by row into training, validation, and test sets. It should be specifically noted that this division is primarily used for estimating the out-of-bag (OOB) error within the model and for robustly calculating feature importance, rather than for evaluating the model’s ability to generalize to external new data. The contribution of each predictor variable to product appeal is quantified using two feature importance assessment methods: Mean Decrease Accuracy (MDA) and Mean Decrease Impurity (MDI). The contribution of each predictor to product attractiveness was quantified using two feature importance assessment methods: Mean Decrease in Accuracy (MDA) and Mean Decrease in Impurity (MDI).

MDA assesses the importance of a feature by measuring the decline in model performance (e.g., predictive accuracy) after the values of that single feature are randomly permuted. A larger decrease in performance indicates a more important feature. The permutation importance based on RMSE can be defined as:

(6) $${Importance}_{j}={RMSE}_{permuted,j}-{RMSE}_{original}$$

where ${RMSE}_{original}$ is the root mean squared error of the original model on the test data, and ${RMSE}_{permuted,j}$ is the RMSE on the same test data after the values of feature *j* have been randomly permuted.

MDI measures a feature’s effectiveness in partitioning the data when constructing the decision trees within the model. It evaluates feature importance based on the reduction in impurity (such as Gini impurity or information entropy) contributed by the feature in the decision trees. For each feature, the MDI can be calculated as:

(7) $${MDI}_{j}=\sum_{t=1}^{T}\underset{i\in t}{\sum }\frac{n_{i}}{N}\Delta I(t,j)$$

where ${MDI}_{j}$ is the mean decrease in impurity for feature *j*, *T* is the total number of trees in the forest, *t* is a specific tree in the forest, $n_{i}$ is a node in tree *t*, *N* is the total number of samples in the training data, and ΔI(t,j) is the reduction in impurity caused by feature *j* at node i of tree *t*.

## 4. Results

### 4.1. Expert Questionnaire: Product Attribute Evaluation

**Figure 6:**
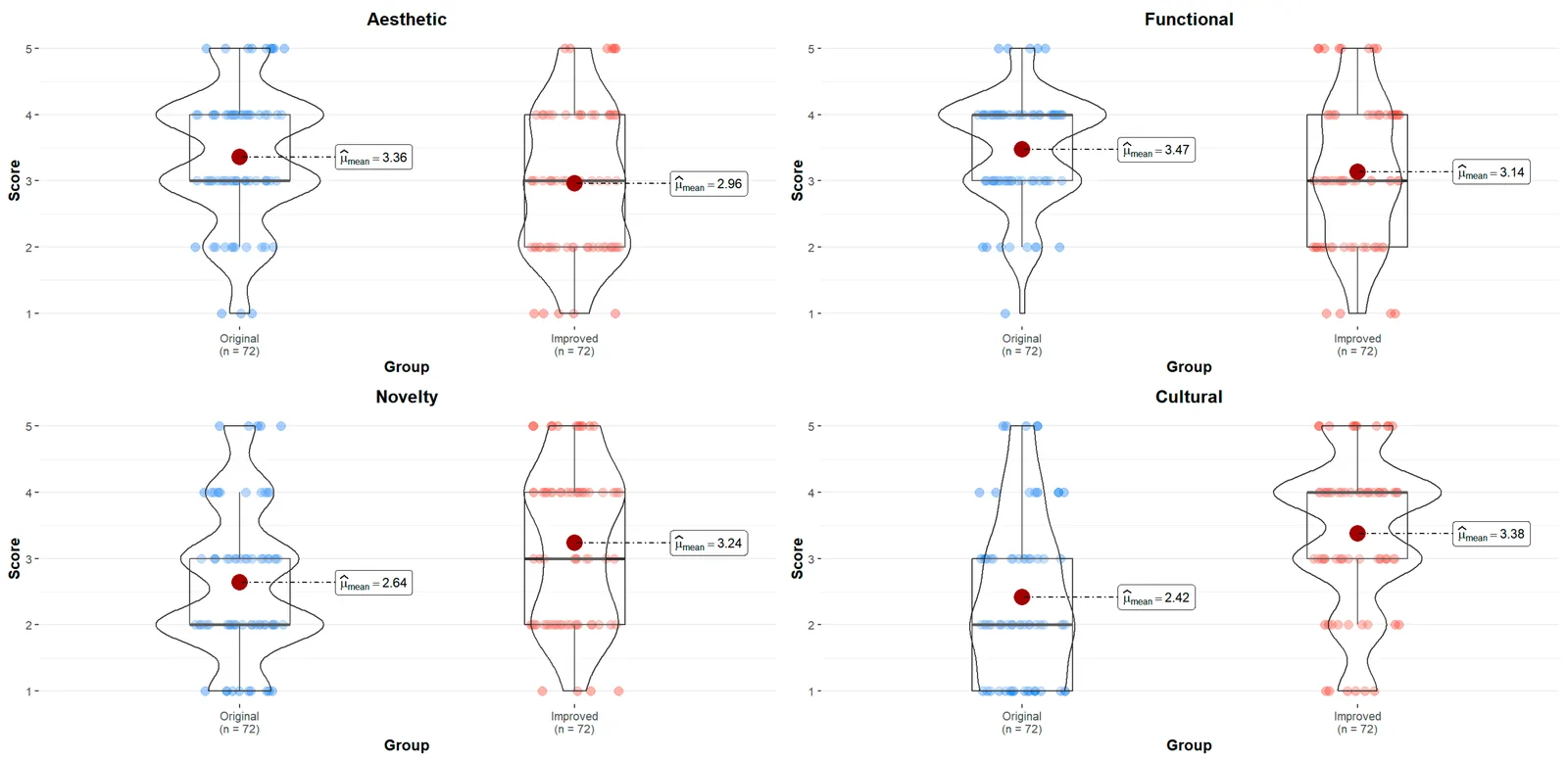
Expert evaluation distribution of original vs. improved products across four product attribute dimensions (aesthetic, functional, novelty, cultural).

The Cronbach’s α coefficient was 0.816 (>0.8), indicating the high internal consistency of the scale. The Intraclass Correlation Coefficient (ICC) for the experts was 0.509, showing a moderate-to-good level of inter-rater reliability. Therefore, the evaluation results were deemed reliable and valid. Table 6 lists the means and standard deviations of the eight products across the four dimensions of aesthetic quality, functionality, novelty, and culture attribute. These data were used for subsequent analyses of group differences and influence mechanisms, and the overall distribution of expert evaluations is visualized in Figure 6. 

### 4.2. Eye-Tracking Data: Preprocessing and Factor Extraction

#### 4.2.1. Data Preprocessing

**Figure 7:**
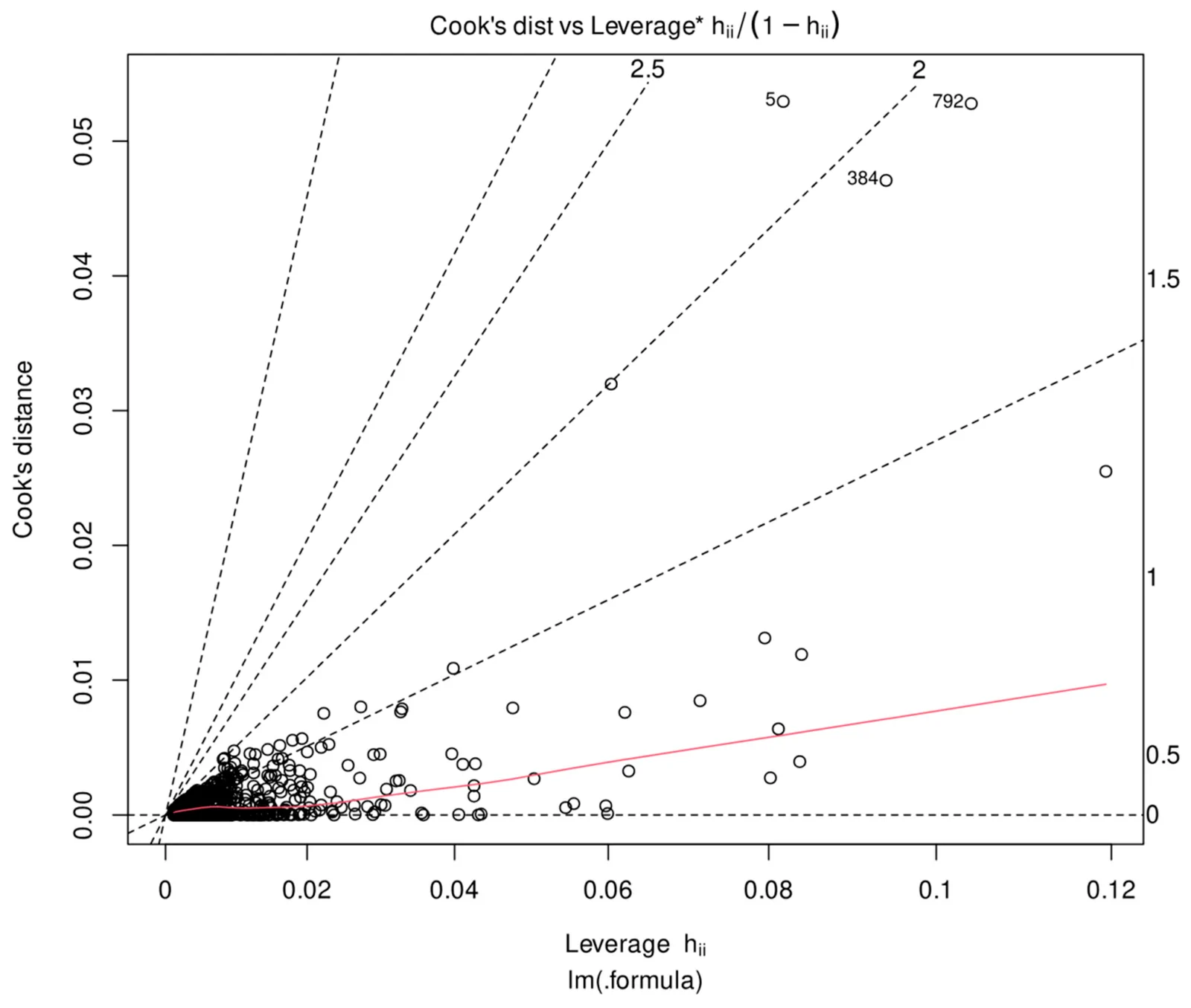
Cook’s distance vs. leverage plot for the linear regression model. The horizontal axis represents leverage, and the vertical axis represents Cook’s distance. The contour lines in the scatter plot indicate standardized residuals, labeled with their magnitudes.

The raw eye-tracking dataset was extracted from the 146 participants who were retained after screening for a valid sampling rate (>70%), comprising a total of 1168 records and 8183 data points from these 146 participants. First, 42 invalid records containing NaN values were removed. The remaining 1126 records were then standardized using Z-scores. Subsequently, a linear regression analysis was performed with the seven eye-tracking metrics and product attractiveness scores, and outliers were identified using Cook’s distance and leverage values (Figure 7). The results showed that while the Cook’s distances for most observations were minimal, they were significantly elevated for observations 5, 191, 384, 651, and 792 (all > 0.02). Further inspection revealed that points with high Cook’s distances often had higher leverage values, with observation 191 exhibiting the highest leverage, indicating it was an extreme point in the independent variable space. The literature suggests that this phenomenon may be caused by participants closing their eyes or shifting their gaze away during the experiment [83]. Given that recent studies on eye-tracking data and product evaluation have used Cook’s distance (with a threshold set at >0.02) to identify and exclude outliers [83], and to ensure the rigor and reliability of the analysis, this study ultimately excluded the aforementioned five outliers and retained 1121 valid records for subsequent analysis.

#### 4.2.2. Correlation Analysis of Eye-Tracking Metrics

**Figure 8:**
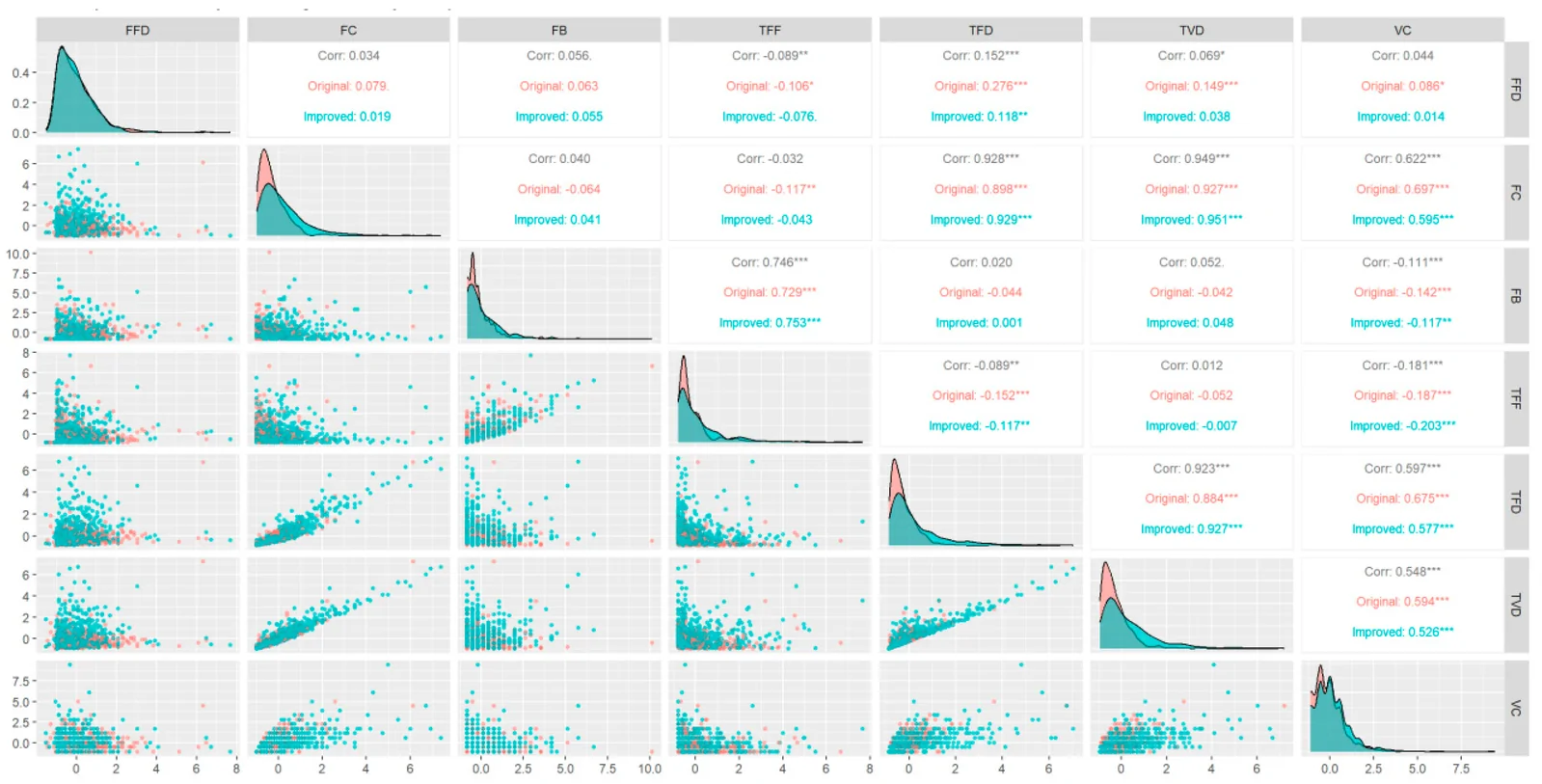
Correlation matrix plot of seven raw eye-tracking metrics for original and improved groups.

To understand the relationships among the metrics, a correlation analysis was conducted on the seven raw eye-tracking metrics (Figure 8). The results were as follows:

(1) Extremely high positive correlations were found between Fixation Count and Total Fixation Duration (*r* = 0.928), Fixation Count and Total Visit Duration (*r* = 0.949), and Total Fixation Duration and Total Visit Duration (*r* = 0.923). These near-unity correlation coefficients indicate a high degree of overlap among these three metrics.
(2) A strong correlation was observed between Fixation Before and Time to First Fixation (*r* = 0.746), suggesting that these two metrics may reflect similar underlying information to some extent.
(3) Additionally, strong correlations were also present between Fixation Count and Visit Count (*r* = 0.622), Total Fixation Duration and Visit Count (*r* = 0.597), and Total Visit Duration and Visit Count (*r* = 0.548).

These findings indicate the presence of strong linear relationships among multiple raw eye-tracking metrics. Therefore, it was necessary to extract a few relatively independent principal components (eye-tracking factors).

#### 4.2.3. Principal Component Analysis and Eye-Tracking Factor Extraction

**Figure 9:**
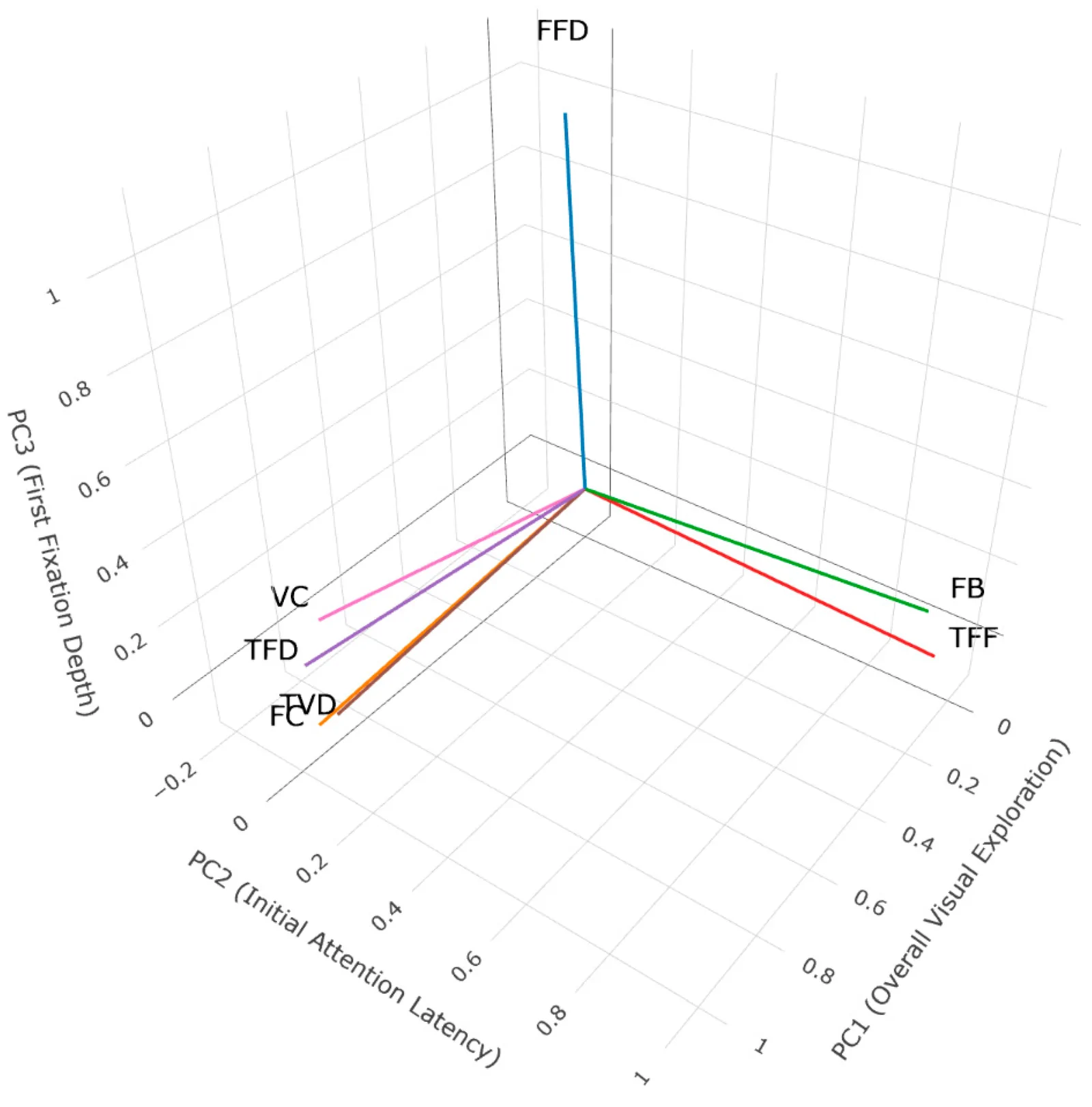
3D variable loading plot of the rotated PCA. This plot displays the loading vectors of the seven raw eye-tracking metrics on the three principal components after varimax rotation. As the figure shows, the variables are clearly grouped into three clusters based on their function: metrics like FC (loading = 0.974) and TFD (loading = 0.956) align almost entirely along the PC1 axis, collectively defining “Overall Visual Exploration”; FB (loading = 0.931) and TFF (loading = 0.931) are highly aligned with the PC2 axis, defining “Initial Attention Latency”; while FFD (loading = 0.996) independently constitutes the PC3 axis, or “First Fixation Depth,” visually validating the effectiveness of the factor structure.

Principal Component Analysis (PCA) was employed to reduce the dimensionality of the seven raw eye-tracking metrics. The analysis used Varimax rotation with Kaiser Normalizatio (Figure 9)n. The results (Table 7) revealed three factors with eigenvalues greater than 1, which collectively explained 87.421% of the total variance. Specifically:

(1) The first principal component was composed of Fixation Count (0.974), Total Fixation Duration (0.956), Total Visit Duration (0.955), and Visit Count (0.733). As these metrics primarily represent the total amount or intensity of visual engagement, this factor was named “Overall Visual Exploration”.
(2) The second principal component consisted of Fixation Before (0.931) and Time to First Fixation (0.931). Both metrics are related to behavior at the onset of observation. A higher score on this component signifies a slower speed of attention orientation (i.e., a longer delay before initial engagement). It was therefore named “Initial Attention Latency”.
(3) The third principal component was almost exclusively defined by First Fixation Duration (0.996), reflecting the duration of initial information processing. It was accordingly named “First Fixation Depth”.

In conclusion, the extraction of these eye-tracking factors helps to reveal underlying patterns of visual behavior and significantly enhances the efficiency, stability, and interpretability of subsequent research.

### 4.3. User Questionnaire: Overall Product Attractiveness and Classification into Relative Attractiveness Tiers

For the user questionnaire, the Cronbach’s α was 0.819, indicating high internal consistency. The overall distribution of attractiveness scores was slightly right-skewed (skewness = 0.35). Based on this, the parameters for the triangular fuzzy numbers were determined, and the membership degrees for each product’s attractiveness were calculated. The products were then classified into relative tiers according to the principle of maximum membership degree: ID:3 and ID:5 were classified as Tier 3; ID:1, ID:2, ID:6, and ID:7 as Tier 2; and ID:4 and ID:8 as Tier 1. From the user’s perspective, the results indicate that the aesthetic ratings of the improved products were enhanced to varying degrees compared to the original products (Table 8).

### 4.4. Comparative Analysis of Original and Improved Products

#### 4.4.1. Paired Nonparametric Comparison

This study employed a within-subjects design, in which the same group of experts (N = 18) and users (N = 146) evaluated all products; therefore, the data did not satisfy the assumption of independence. Furthermore, the Shapiro–Wilk test revealed that the distribution of differences in some data (eye-tracking data) deviated significantly from a normal distribution. Therefore, the Wilcoxon Signed-Rank Test was uniformly applied to analyze all paired differences (Table 9).

(1) **Expert Questionnaire:** The improved product received significantly higher ratings on the dimensions of novelty and cultural relevance (novelty: *z* = −2.499, *p* = 0.013, *r*_rb_ = −0.733; cultural relevance: z = −2.911, *p* = 0.004, *r*_rb_ = −0.804). On the aesthetics and functionality dimensions, ratings showed a downward trend, but none of these differences reached the preset level of statistical significance (aesthetics: *z* = 1.887, *p* = 0.062, *r*_rb_ = 0.537; functionality: *z* = 1.939, *p* = 0.053, *r*_rb_ = 0.551). This suggests that from an expert’s perspective, while the design improvements enhanced the products’ novelty and culture attribute to some extent, they were insufficient in elevating their aesthetic and functional qualities.
(2) **Eye-Tracking Factors:** The results of the normality test indicated that the difference data for all three eye-tracking factors deviated significantly from a normal distribution. Therefore, it was necessary to use the Wilcoxon signed-rank test. The results show that the improved product scored significantly higher than the original product on both the Overall Visual Exploration and Initial Attention Latency factors (Overall Visual Exploration: *z* = −9.115, *p* < 0.001, *r*_rb_ = −0.870; Initial Attention Latency: *z* = −2.724, *p* = 0.006, *r*_rb_ = −0.260). However, no significant difference was observed between the two groups for First Fixation Depth (*z* = 0.614, *p* = 0.540).
(3) **User Evaluation Questionnaire:** The Attractiveness scores for the improved group were significantly higher than those for the original group, and the difference was highly consistent (*z* = −6.302, *p* < 0.001, *r*_rb_ = −0.838). This demonstrates that the paper-cutting products designed using the SCAMPER-based approach achieved a significant improvement in user evaluations compared to the original products, thereby providing indirect validation for the effectiveness of the design method.

**Table 9:** Results of Wilcoxon signed-rank test for paired comparisons between original and improved groups.

Category	Variable	*z*	*p*	Rank-Biserial Correlation (*r*_rb_)
Product Attributes	Aesthetic	1.887	0.062	0.537
	Functional	1.939	0.053	0.551
	Novelty	−2.499 *	0.013	−0.733
	Cultural	−2.911 **	0.004	−0.804
Eye-Tracking Behaviors	Ovl.Vis.Exp.	−9.115 ***	<0.001	−0.870
	Ini.Att.Lat.	−2.724 **	0.006	−0.260
	First Fix.Dep.	0.614	0.540	0.059
User Attractiveness	Attractiveness	−6.302 ***	<0.001	−0.838

Note: * *p* < 0.05, ** *p* < 0.01, *** *p* < 0.001. A positive *t*-value indicates that the mean of the original group is higher than that of the improved group, while a negative *t*-value indicates that the mean of the improved group is higher than that of the original group.

#### 4.4.2. Exploratory Analysis of Attractiveness Tier Distribution

To observe the impact of the design intervention on the distribution of user preferences from a macro perspective, we conducted an exploratory analysis of the distribution of the two product groups across the three relative tiers: Tier 1, Tier 2, and Tier 3. As shown in Table 10, ratings for the original group were concentrated in Tier 3 (49.5%) and Tier 2 (50.5%); whereas the improved group completely eliminated Tier 3 (0%) and distributed its ratings evenly between Tier 2 (50%) and Tier 1 (50%). It can be concluded that the improvement measures resulted in a significant structural change in the relative ranking of products within the sample pool, specifically, a shift from lower tiers to higher tiers.

### 4.5. Exploratory Analysis of the Product Attractiveness Correlation Model

#### 4.5.1. Hierarchical Multiple Regression Analysis

To explore how product attributes and eye-tracking metrics jointly influence users’ perception of a product’s attractiveness (RQ3), this study employed an OLS hierarchical regression model with cluster-robust standard errors (CRSEs), using participant ID (Subject) as the clustering variable. The aim of this section is to uncover associative trends within a specific dataset rather than to construct a causal predictive model with generalized applicability. This analysis comprises two models (Table 11):

• **Model 1 (Baseline Model):** Aimed to test hypothesis H3a, i.e., whether product attributes could significantly influence overall product attractiveness as perceived by users.
• **Model 2 (Full Model):** Added the eye-tracking factors to the baseline model to test hypothesis H3b, i.e., whether visual behavior patterns could explain additional variance in attractiveness after controlling for product attributes.

(1) **Baseline Model:** The mathematical expression for this model is:

(8) $$Ai=\beta_{0}+\beta_{1}*(aesthetic)+\beta_{2}*(functional)+\beta_{3}*(novelty)+\beta_{4}*(cultural)+\varepsilon_{0}$$

where *β*_0_ is the intercept, *β*_1_ to *β*_4_ are the coefficients for the respective independent variables, and *ε*_0_ is the error term.

**Figure 10:**
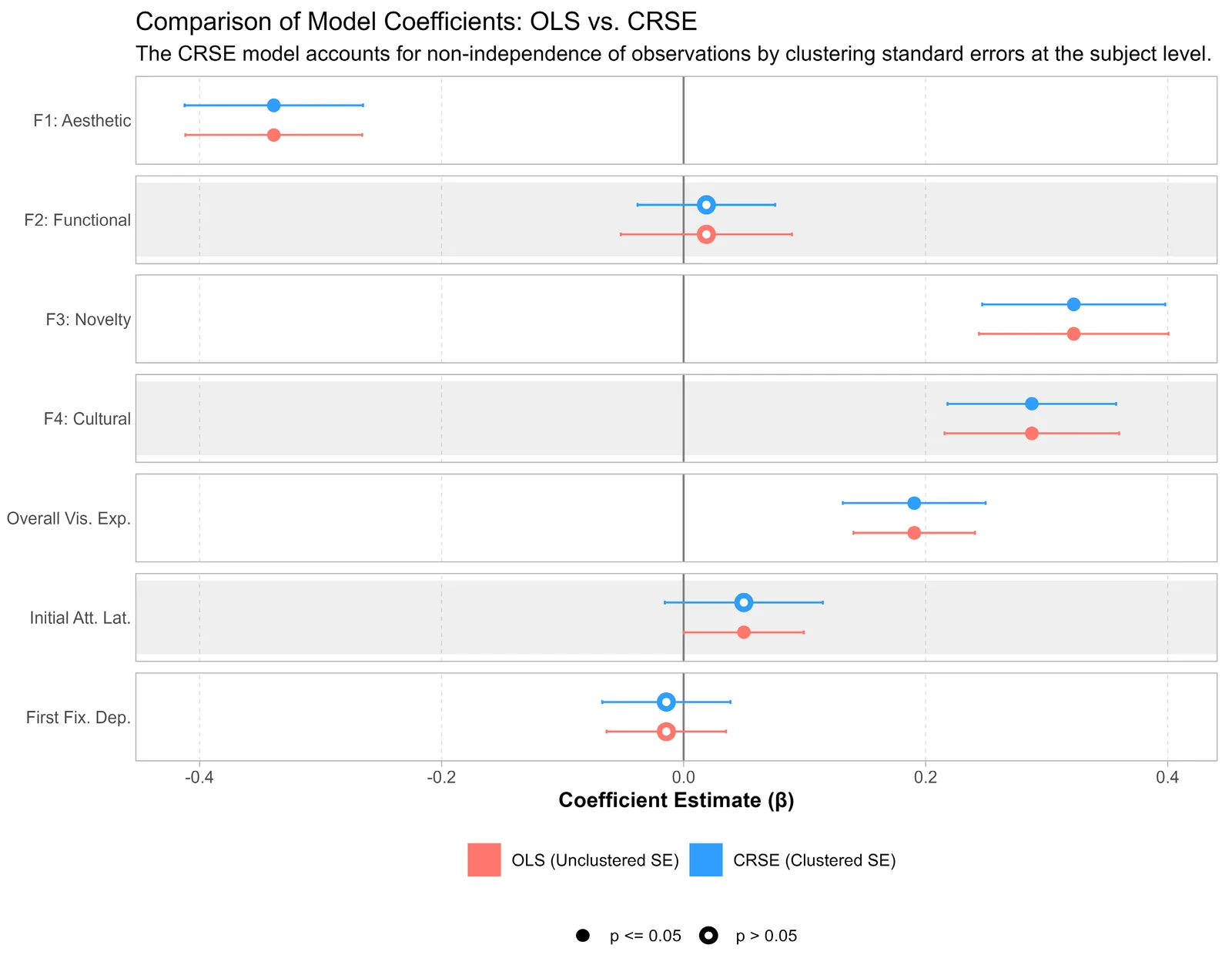
Visual comparison of coefficient estimates and 95% confidence intervals between standard OLS and CRSE models. As can be seen from the figure, the point estimates (*β*) under both specifications are identical. Following the application of CRSE: (1) The confidence intervals (horizontal lines) have generally widened, indicating that the uncertainty in the estimates has increased following the consideration of data non-independence. (2) The significance of one variable has changed: the effect of Initial Attention Latency was significant in the OLS estimates (red solid circle), but following the more stringent CRSE correction, its confidence interval spans zero, rendering it non-significant (blue hollow circle).

The results showed that this model was able to explain 26.3% of the total variance in attractiveness (*R*^2^ = 0.263, Adj. *R*^2^ = 0.260), supporting hypothesis H3a and suggesting that product attributes form the basis of attractiveness judgments. Specifically, after adjusting for cluster-robust standard errors (CRSEs), the coefficients estimated by the model indicate that the estimated coefficients revealed that Novelty (*β* = 0.365, *p* < 0.001) and Culture (*β* = 0.299, *p* < 0.001) were the strongest positive predictors. Unexpectedly, Aesthetic Quality (*β* = −0.353, *p* < 0.001) showed a significant negative association, while Functionality (*β* = −0.006, *p* = 0.873) had no significant effect on attractiveness. Compared with the uncorrected OLS results, the CRSE correction did not alter the conclusions regarding the significance of any variables in the baseline model, but it provided more robust statistical support for these findings (Figure 10). The tolerance values for all predictor variables were greater than 0.404 (VIF < 2.478), indicating that the model did not suffer from severe multicollinearity issues.

(2) **Full Model:** The mathematical expression for this model is:

(9) $$A_{i}=\beta_{0}^{\prime }+\beta_{1}^{\prime }\cdot (aesthetic)+\beta_{2}^{\prime }\cdot (functional)+\beta_{3}^{\prime }\cdot (novelty)+\beta_{4}^{\prime }\cdot (cultural)\;+\beta_{5}^{\prime }\cdot (Ovl.Vis.Exp.)+\beta_{6}^{\prime }\cdot (Ini.Att.Lat.)+\beta_{7}^{\prime }\cdot (1st.Fix.Dep.)+\epsilon_{1}$$

The results showed that the model’s explanatory power increased to 30.0% of the total variance in attractiveness (*R*^2^ = 0.300), with a net *R*^2^ increase of 3.7% (Δ*R*^2^ = 0.037), confirming hypothesis H3b: after controlling for subjective product attributes, users’ objective visual behavior patterns have additional, independent predictive value for product attractiveness.

In the full model, the effect pattern of the product attributes remained largely stable. After adjusting for cluster-robust standard errors, among the newly added eye-tracking metrics, Overall Visual Exploration (*β* = 0.191, *p* < 0.001) remained a significant positive predictor. It is worth noting that Initial Attention Latency, which was marginally significant in the original model, became statistically insignificant after more rigorous correction, with a *p*-value of 0.121. First Fixation Depth (*β* = −0.014, *p* = 0.570) was not significant (Table 12).

In summary:

(1) Product attributes (especially novelty, culture attribute, and aesthetic quality) form the basis of attractiveness judgments (supporting H3a).
(2) Users’ visual attention processes (overall visual exploration) provide significant predictive information for attractiveness (supporting H3b).
(3) The negative effect of aesthetic quality warrants further investigation.

#### 4.5.2. Random Forest Regression

This study constructed a Random Forest Regression (RFR) model, as a nonparametric exploratory tool, to investigate the nonlinear relationships and interaction effects between product attributes and eye-tracking metrics on product attractiveness ($A_{i}$). The data were partitioned into a training set (*n* = 717), a validation set (*n* = 180), and a test set (*n* = 224). It should be noted that, given this study focuses on eight specific combinations of product attributes, the random division employed here is primarily intended for controlling the in-sample out-of-bag error and assessing feature importance, rather than for external generalization to entirely new product categories. Hyperparameters were set to n_estimators = 99 and max_features = 2, with out-of-bag MSE as the optimization target. The performance evaluation of the RFR model showed that the test set MSE (0.621) was close to the validation set MSE (0.611), indicating stable in-model fit. The test set RMSE was 0.788, and the model ultimately explained 34.0% of the variance in product attractiveness (*R*^2^ = 0.340), outperforming the CRSE-corrected hierarchical linear regression model (OLS, *R*^2^ = 0.300). This suggests that the RFR model may have more effectively captured the complex relationships between the variables (Table 13).

A feature importance analysis was conducted to reveal the contribution of each predictor to the model’s predictive accuracy. According to the MDA metric:

(1) Among the product attributes, Novelty (MDA = 0.271) and Culture Attribute (MDA = 0.243) were the two most important associated factors. Aesthetic Quality (F1-aesthetic-z) (MDA = 0.101) ranked third, indicating it is equally crucial for assessing attractiveness.
(2) Functionality (F2-functional-z) (MDA = 0.094) and the eye-tracking factor Overall Visual Exploration (Ovl.Vis.Exp., MDA = 0.087) constitute the intermediate tier of explanatory power.
(3) The eye-tracking factor Initial Attention Latency (Ini.Att.Lat., MDA = 0.040) had a lower contribution, while First Fixation Depth (1st.Fix.Dep., MDA = −0.010) had a negative importance value, suggesting it made no contribution to the model’s predictions.

The MDI metric provided a complementary perspective. Under this metric, the importance rankings of the eye-tracking factors “Overall Visual Exploration” and “Initial Attention Latency” rose, surpassing those of “Aesthetic Quality” and “Functionality.” This implies that visual behavior patterns have intrinsic value in distinguishing product attractiveness (Table 14).

## 5. Discussion

### 5.1. Addressing RQ1: On the PPI-SCAMPER Method

In response to the research question (RQ1) of how to effectively integrate SCAMPER with traditional paper-cutting aesthetics, this study developed the PPI-SCAMPER method. This method transforms abstract thinking techniques into a structured design support tool, which demonstrated positive effects in its application. Concurrently, we identified three issues worthy of discussion.

First, the choice of the initial design path: Despite the introduction of material libraries and a fixed process, designers still face a choice between a “product-form-driven” or a “paper-cutting-pattern-driven” approach. The former is suitable for goal-oriented, incremental improvements, while the latter is more likely to generate disruptive concepts in exploratory design. This suggests that future iterations of PPI-SCAMPER could be refined to offer differentiated application paths based on the task type. At the same time, while the openness of the current path provides flexibility, it also places higher demands on the designer’s comprehensive skills.

Second, the subjective ambiguity of concept evaluation: To address the issue of subjectivity in screening concepts after SCAMPER-based divergence, future research should focus on developing computational models for decision support, providing designers with objective evaluation references. For example, developing a design fixation computational framework (such as BF-AIS) could facilitate the transition from traditional manual assessment to the automated detection of design fixation [112]. This, in turn, relies on conducting more in-depth laboratory research on design fixation to serve as foundational support, such as decoding the relationship between designer behavior and fixation [113].

Third, the value of analogical distance: The literature has clearly established that selecting an appropriate analogical distance is key to stimulating innovation; analogies that are too close can limit creativity [6], while those that are too distant may lead to infeasible solutions [7]. In the application of our method, we observed that designers’ use of SCAMPER operators implicitly involves a choice of analogical distance. For example, in the fan case study, selecting the “Hollow out” process, which is functionally close to ventilation, represents a near-field functional analogy, often corresponding to milder operators like “Substitute” or “Combine”. In contrast, operators like “Put to other uses” or “Reverse” have a more disruptive, far-field character. Further research has confirmed that near-field analogies are used more frequently and result in higher-quality concepts, whereas far-field analogies significantly enhance concept novelty [114]. Therefore, further exploring and quantifying the value of analogical distance may offer a shortcut (a feasible operation) to help designers achieve their specific innovation goals, whether incremental or breakthrough. This provides valuable insights for the future development of analogical design support tools [115].

### 5.2. Addressing RQ2: A Comprehensive Evaluation of the PPI-SCAMPER Method’s Effectiveness

In addressing RQ2, a synthesis of the *t*-test and chi-square test results (see Section 4.4) leads to a complex yet insightful conclusion: the improved designs achieved overwhelming success at the user level, but revealed an intriguing trade-off at the expert level.

From the user’s perspective, the effects of the improved designs were unequivocally positive. The subjective attractiveness scores showed that the attractiveness of the improved group was enhanced to a highly significant degree (*z* = −6.302, *p* < 0.001, *r*_rb_ = −0.838). This enormous effect size provides powerful evidence of the success of the improvement strategy in appealing to popular aesthetic preferences. Objective eye-tracking data, however, painted a more nuanced picture. The improved products significantly elicited more extensive visual exploration from users (*z* = −9.115, *p* < 0.001, *r*_rb_ = −0.870). This may indicate that the incorporation of paper-cutting art has successfully enhanced the visual richness and appeal of the product, or it may suggest that the higher visual complexity has caused confusion amongst the test subjects or made the search more difficult. Nevertheless, in terms of the speed of capturing users’ initial attention, the improved group actually exhibited a longer delay (*z* = −2.724, *p* = 0.006, *r*_rb_ = −0.260). Does this imply that more complex visual elements might increase the cognitive processing load at first sight, requiring more time to locate and identify key areas? Despite this, an exploratory analysis of the distribution of attractiveness across different tiers ultimately confirmed the overall positive results, showing that the improvements completely eliminated low attractiveness ratings and significantly shifted product evaluations into the medium-to-high attractiveness range.

The expert evaluations, however, were more complex. The improved products were significantly superior to the original ones in terms of novelty (*z* = −2.499, *p* = 0.013) and cultural aspect (*z* = −2.911, *p* = 0.004), which aligns perfectly with the PPI-SCAMPER method’s aim to integrate cultural elements and stimulate innovation. Yet, unexpectedly, the expert ratings for the two traditional design dimensions of aesthetic quality (*p* = 0.062) and functionality (*p* = 0.053) both decreased (however, none of these differences reached the pre-specified level of statistical significance). Previous research has repeatedly pointed out that there is an asymmetry between expert and user evaluations [17,18]. One possible explanation for this is that experts’ evaluation criteria may place greater emphasis on classical aesthetic principles and formal purity. While novel paper-cutting elements may offer cultural impact and uniqueness, they may, in the eyes of experts, disrupt the product’s inherent formal harmony or the directness of its functional expression. In contrast, ordinary users may be more easily drawn to novel visual stimuli and cultural narratives, and may be less sensitive to deviations from traditional aesthetic norms.

In summary, the PPI-SCAMPER method significantly enhanced the products’ sense of innovation, cultural connotation, and ultimate user attractiveness, but possibly at the expense of the traditional aesthetic and functional purity valued by experts. This finding points to a classic trade-off in innovative design: the tension between uniqueness and classicism, which predicts the combined effect of these two seemingly opposing forces on aesthetic appreciation [116]. The deep relationship between this trade-off and analogical design warrants further investigation.

### 5.3. Addressing RQ3: Investigating the Association Patterns of Product Attractiveness

The third research question (RQ3) of this study aims to explore the patterns of association among product attributes, users’ visual behavior and product appeal. By integrating the results of hierarchical regression adjusted for cluster-robust standard errors (CRSEs) and Random Forest models, we not only validated our research hypotheses but, more importantly, these findings prompted a deeper reflection on existing research paradigms and counter-intuitive phenomena.

First, the results confirm that product attributes form the foundation of attractiveness judgments, while physiological metrics provide only a limited incremental benefit. The hierarchical regression showed that the baseline model, composed of product attributes, explained 26.3% of the variance (supporting H3a). The fact that this explanatory power did not reach higher levels can be attributed to two main factors: first, perceived attractiveness is inherently subjective and may involve not only product features but also personal tastes, educational experiences, and cultural values [33]; and second, the inherent cognitive gap between expert evaluations and user perceptions limits the predictive ceiling [17,18,117]. We posit that the latter may be the more dominant reason. Building on this, the introduction of eye-tracking factors increased the model’s explanatory power by only 3.7% (supporting H3b). This limited increment warrants reflection: some studies tend to overemphasize the predictive power of physiological data (such as eye tracking) while overlooking the product attributes that are closely tied to the designer’s intent. As research has pointed out, eye-tracking technology itself cannot directly reveal any information unless its intrinsic associations are correctly understood [118]. Buchanan argued that a designer is, in effect, creating a persuasive argument [119], and the product attributes are the very substance of that argument and the basis for user judgment. Therefore, the 3.7% increment from eye-tracking data may simply indicate that, on top of the strong foundation of product attributes, the contribution of visual behavior is naturally limited. However, this inference awaits further empirical validation or replication.

Second, the negative predictive effect of “aesthetic quality” (*β* = −0.339, *p* < 0.001 in Model 2) is a paradox worth dissecting. This contradicts Crilly’s definition [21], where an “aesthetic impression may be defined as the sensation that results from the perception of attractiveness (or unattractiveness) in products.” A possible explanation is the cognitive discrepancy between experts and users: the “aesthetic quality” valued by experts, which may emphasize classicism and harmony, is not equivalent to the “attractiveness” preferred by users, which may favor novelty and interest. This could lead to the former becoming a negative predictor for the latter. Additionally, other potential reasons include complex interaction mechanisms, where aesthetic quality might influence attractiveness through its interplay with other attributes (such as novelty) in ways that are beyond the capture of a linear model.

### 5.4. A Comprehensive Reflection: On the Dichotomy of Results

Our findings appear to present a stark dichotomy: if the integration of cultural elements into modern products is viewed as an advantage (as is the case for the majority of users, who provided positive feedback), the design is deemed significantly improved. Conversely, if it is viewed as a disadvantage (as is the case for the experts, for whom novelty and culture attribute exceeded the original products, yet aesthetic quality and functionality declined significantly), the design is considered relatively weak. Does this imply that PPI-SCAMPER is not a comprehensive method, and that the enhancements in product novelty and culture attribute are achieved at the expense of aesthetics and functionality? This issue warrants careful reflection:

First, this phenomenon should not be simply attributed to a flaw in the method itself. On the one hand, user evaluations of the improved designs’ attractiveness (both in overall scores and level distribution) comprehensively outperformed the original designs, which already validates the success of the method. On the other hand, the expert data precludes the simplistic explanation that ratings were blindly driven by the mere presence of paper-cutting elements or influenced by design quality. Although the overall nonparametric paired test (Wilcoxon signed-rank test) showed a significant increase in novelty and culture attribute for the improved group, an examination of individual cases reveals that in the first paired comparison, the novelty score of the improved product (ID:2, 2.50) was actually lower than that of the original product (ID:1, 2.94). Coupled with the Intraclass Correlation Coefficient (ICC = 0.509), this proves that experts did not merely award high scores because paper-cutting elements were present; rather, they carefully considered the overall effect and integration techniques. Therefore, the evaluation discrepancy is more accurately a profound empirical manifestation of the cognitive asymmetry within the “designer’s intent vs. user’s interpretation” model, which has been documented by numerous instances in previous design research [17,18,98] and revealed through methods such as semantic differential studies of morphological perception [120].

Second, given that this asymmetry objectively exists, the ensuing question is: Can it be managed or reconciled? To address such issues, there is a greater need to help designers balance and support diverse meanings, rather than merely imposing a single authoritative interpretation [121]. From a more operational standpoint, we argue that implementing innovation goal management, while acknowledging the classic tension between “novelty” and “typicality” (e.g., the MAYA principle [116]) in innovative design, might be a viable path. For instance, within the framework of analogical design, by quantifying and controlling the “analogical distance,” designers can strategically opt for incremental innovation (near-field analogy) or breakthrough innovation (far-field analogy) [114], thereby bringing this cognitive divergence into the realm of controllable design management.

Third, starting from specific product attributes, future applications must expand into deeper dimensions to address the shortcomings in functional and aesthetic ratings observed in this study. Regarding functionality, a potential enhancement approach is that the utilization and analogy of traditional paper-cutting elements must transcend pure surface styling and move towards Design Morphology [122], for example, extending this to structural mechanics (e.g., the application of origami/kirigami structures in engineering) [123,124] or cross-disciplinary inspiration from material properties [125], thereby enabling cultural elements to yield substantial physical benefits. Regarding aesthetic quality, given the fundamental differences in recognition between experts and users, introducing participatory co-creation early in the design process is a viable avenue for seeking balance. The insufficient scores on certain factors highlight the need for us to conduct interdisciplinary research.

### 5.5. Limitations and Future Research

In terms of methodology, while the effectiveness of PPI-SCAMPER has been validated, key decision points within the method—such as the choice of design driver and the subjectivity of evaluation—still require optimization. Future work could involve developing more refined sub-processes or auxiliary decision-making models to enhance its operability and objectivity. Furthermore, exploring and quantifying analogical distance is an effective pathway to assist designers in achieving their specific innovation goals, whether incremental or breakthrough. This also offers valuable insights for the development of analogical design support tools. A further avenue for future research includes leveraging artificial intelligence (AI) to construct intelligent analogical design support tools, and even computational co-creative systems [11,126].

Regarding variable measurement, although the expert ratings underwent reliability checks, they remain a subjective measure. Specific features of individual product designs may exert a significant impact on the final evaluation outcomes. Consequently, future research should further enhance the objective analysis of form during the design development phase. For instance, computational aesthetic algorithms could be incorporated to objectively quantify a product’s formal complexity or symmetry, thereby more precisely controlling and isolating the independent effects of specific design features on perception.

The experimental stimuli in this study were limited to four pairs of product images (eight in total), which is a relatively small number. Although the research focused on innovative product development integrating paper-cutting art, the types and number of products should be further expanded in the future. Concurrently, the user participants in this study were primarily university students (both undergraduate and postgraduate) from a similar cultural background. Future research should broaden the sample to include a more diverse audience with varying ages, occupations, and cultural backgrounds.

At the theoretical level, this study revealed the subordinate role of visual factors and the counter-intuitive effects of “aesthetic quality,” for which possible explanations were proposed. However, these explanations still require more targeted experimental designs for validation.

The cross-validation strategy employed for the Random Forest analysis in this study was designed to explore patterns within the sample rather than to construct a predictive model with external generalization capabilities. Future research seeking a more unbiased assessment of feature importance may adopt more complex validation methods based on the data hierarchy.

## 6. Conclusions

This study aims to develop an innovative method capable of systematically integrating traditional paper-cutting art into modern product design, to construct and validate a full research framework from design generation to multi-dimensional evaluation, and thereby to deeply unravel the perceptual discrepancies between users and experts, as well as the formation mechanisms of attractiveness for such innovative products. By developing the PPI-SCAMPER design method and combining it with expert questionnaires, user questionnaires, and eye-tracking experiments, this study has arrived at the following core conclusions:

In terms of methodology (addressing RQ1), this study successfully developed a structured design support tool called PPI-SCAMPER. By establishing a knowledge base to guide creative ideation, incorporating built-in evaluation to optimize concept screening, and formalizing the process to enhance systematic application, this method effectively addresses the limitations of the traditional SCAMPER method in specific design tasks.

Design Effectiveness (addressing RQ2): The products improved via the PPI-SCAMPER method achieved considerable success at the user level, with their user attractiveness and visual exploration being significantly higher than those of the original products. However, this success manifested as a complex trade-off at the expert level: while the products showed significant improvements in novelty and cultural aspect, they declined in the traditional dimensions of aesthetic quality and functionality valued by experts (although none of these differences reached the preset level of statistical significance). Nevertheless, this finding may reveal an inherent tension between “uniqueness” and “classicism” in innovative design and warrants further investigation.

Association Patterns (addressing RQ3): Product attributes (especially novelty and cultural aspect) form the foundation of attractiveness judgments, while users’ objective visual behaviors provide valuable additional explanatory information on top of that. Notably, this study identified a counter-intuitive phenomenon: experts’ aesthetic evaluations showed a significant negative correlation with users’ perceptions of attractiveness, reflecting a cognitive barrier between experts and users and empirically demonstrating aspects of the “intent–interpretation” model [17,18].

In summary, this study not only proposes a practical method for integrating traditional cultural elements from an analogical design perspective but also provides theoretical insights for understanding how innovative products are perceived and evaluated through multi-dimensional empirical data, thereby opening up new directions for future research. Although the generalizability of this study’s conclusions requires further testing (as the user sample consisted primarily of students with a background in design), the “expert–user” cognitive divergence model revealed in the study, and the potential demonstrated by the PPI-SCAMPER framework as a case study in the revitalization of local cultural elements, remain highly instructive. On the one hand, if the application of Chinese paper-cutting in this study is viewed as a localized case study for revitalizing decorative elements, the PPI-SCAMPER framework demonstrates the potential for transferability to other local cultural traditions (e.g., Islamic geometric patterns [127]). On the other hand, this paper effectively establishes a quantitative evaluation system that synthesizes multimodal data, paving the way for the scientific assessment of future innovative designs. Finally, we look forward to the future use of traditional elements such as paper-cutting expanding into Design Morphology [122] (e.g., structural engineering, kinematics, and material intelligence). This expansion will transcend the boundaries of cultural product design and heritage-oriented innovation; advance the Design-by-Analogy Method; realize the cross-disciplinary translation of cultural elements; and drive the profound integration of aesthetics, functionality, and meaning.

## Figures and Tables

**Table 1:** Definitions and the relevant literature for product attribute dimensions.

Product Attribute Indicator	Description	References
Aesthetic Quality	Measures whether a product is visually appealing, harmonious, and aesthetically pleasing.	[21,57,58,59,60]
Functionality	Evaluates whether a product is effective, practical, and capable of meeting users’ needs.	[57,64,65,66,67]
Novelty	Considers the uniqueness and innovation of a product’s design, as well as the sense of surprise it brings to users.	[61,64,68,69,70]
Culture Attribute	Examines how a product embodies, conveys, and integrates specific cultural elements and connotations.	[62,63,71,72,73]

**Table 2:** Definitions of eye-tracking metrics.

	Index	Notation
1	Time to First Fixation	Total gaze time before the first entry into AOI
2	Fixation Before	Total number of gaze points before the first entry into AOI
3	First Fixation Duration	Duration of the appearance of the first gaze point in AOI
4	Total Fixation Duration	Sum of the duration of all gaze points in AOI
5	Fixation Count	Sum of the number of all gaze points in AOI
6	Visit Count	Sum of the number of all gaze counts in AOI
7	Total Visit Duration	Sum of all gaze times in AOI

**Table 3:** Definitions and the relevant literature for subjective attractiveness indicators.

Attractiveness Indicator	Description	Reference
Liking	Measures the user’s overall favorability and emotional connection to the product, reflecting their subjective preference.	[86,92,93,94,95]
Recommendation	Measures the user’s willingness to recommend the product to others (friends or colleagues), reflecting their satisfaction and word-of-mouth potential.	[89,91,96,97]

**Table 4:** Demographic information of the expert participants.

Category	Composition
Gender	Male: 38.9% (*n* = 7) Female: 61.1% (*n* = 11)
Age	31–35 years old: 22.2% (*n* = 4) 36–40 years old: 66.7% (*n* = 12) 41–45 years old: 11.1% (*n* = 2)
Education Level	PhD: 33.3% (*n* = 6) Master’s: 50.0% (*n* = 9) Bachelor’s: 16.7% (*n* = 3)
Years of Relevant Experience	5–8 years: 33.3% (*n* = 6) 9–12 years: 55.6% (*n* = 10) 13–14 years: 11.1% (*n* = 2)
Core Areas of Expertise	Interaction Design and UX Design: 44.4% (*n* = 8) Automotive Styling: 33.3% (*n* = 6) Design Educator: 22.2% (*n* = 4)

**Table 5:** Demographic information of the user participants.

Category	Composition
Gender	Male: 32.9% (*n* = 48) Female: 67.1% (*n* = 98)
Age	19–24 years old: 100% (*n* = 146)
Education Level	Postgraduate: 54.1% (*n* = 79) Undergraduate: 45.9% (*n* = 67)
Major	Industrial Design, Product Design: 100% (*n* = 146)

**Table 6:** Means and standard deviations of the eight products across four dimensions: aesthetic, functional, novelty, and cultural.

	F1-Aesthetic		F2-Functional		F3-Novelty		F4-Cultural	
ID	Mean	SD	Mean	SD	Mean	SD	Mean	SD
1	2.944	1.11	2.944	0.802	2.944	1.056	2.667	1.188
2	2.667	0.84	2.389	0.778	2.5	1.043	3.111	1.023
3	3.333	0.84	3.722	0.575	2.111	0.832	1.833	0.924
4	2.833	1.2	3.556	0.922	3.667	1.237	3.667	1.237
5	3.167	0.924	3.444	0.705	2	0.686	2.111	1.132
6	2.778	1.003	2.833	1.098	2.833	1.098	3.111	1.231
7	4	0.97	3.778	1.003	3.5	1.15	3.056	1.259
8	3.556	1.149	3.778	0.943	3.944	0.802	3.611	1.037

**Table 7:** Summary of factor analysis results for eye-tracking indicators using principal component analysis with varimax rotation.

Factor	Name	High-Loading Indicators	Interpretation
1	Overall Visual Exploration	Fixation Count (0.974) Total Fixation Duration (0.956) Total Visit Duration (0.955) Visit Count (0.733)	Represents the total amount or intensity of visual engagement.
2	Initial Attention Latency	Fixation Before (0.931) Time To First Fixation (0.931)	Reflects the speed of initial attention orientation; higher scores mean slower orientation (longer delay).
3	First Fixation Depth	First Fixation Duration (0.996)	Reflects the duration of initial information processing during the first fixation.

**Table 8:** Results of product attractiveness level classification based on triangular fuzzy numbers.

ID	${\overset{¯}{A}}_{i}$	Low Membership $\mu_{i}\left(3\right)$	Medium Membership $\mu_{i}\left(2\right)$	High Membership $\mu_{i}\left(1\right)$	Attractiveness Level	Group
1	1.91	0.44	**0.56**	0.00	Tier 2	Original
2	2.24	0.00	**0.93**	0.07	Tier 2	Improved
3	1.70	**0.78**	0.22	0.00	Tier 3	Original
4	2.91	0.00	0.00	**0.99**	Tier 1	Improved
5	1.70	**0.78**	0.22	0.00	Tier 3	Original
6	2.53	0.00	**0.51**	0.49	Tier 2	Improved
7	2.18	0.02	**0.98**	0.00	Tier 2	Original
8	2.85	0.00	0.04	**0.96**	Tier 1	Improved

Note: Bold values indicate the maximum membership degree.

**Table 10:** Cross-tabulation of product attractiveness level by group.

Group	Tier 3 *n* (%)	Tier 2 *n* (%)	Tier 1 *n* (%)	Total
Original	275 (49.5)	280 (50.5)	0 (0.0)	555 (100.0)
Improved	0 (0.0)	283 (50.0)	283 (50.0)	566 (100.0)
Total	275 (24.5)	563 (50.2)	283 (25.2)	1121 (100.0)

Note: Values represent observed counts (*n*) and row percentages (%). Row percentages indicate the distribution of attractiveness levels within each group.

**Table 11:** Summary of hierarchical regression models (OLS estimates).

Model	*R* ^2^	Adj. *R*^2^	Δ*R*^2^
Model 1	0.263	0.260	0.263
Model 2	0.300	0.296	0.037

**Table 12:** Coefficients of hierarchical regression models predicting product attractiveness (OLS estimates with cluster-robust standard errors).

Model	Predictor	*β*	Std. Error	*t*	*p*
Model 1	(Intercept)		0.016	0.000	1.000
	F1-aesthetic	−0.353 ***	0.037	−9.616	<0.001
	F2-functional	−0.006	0.030	−0.200	0.841
	F3-novelty	0.365 ***	0.037	9.776	<0.001
	F4-cultural	0.299 ***	0.035	8.583	<0.001
Model 2	(Intercept)		0.019	0.000	1.000
	F1-aesthetic	−0.339 ***	0.037	−9.163	<0.001
	F2-functional	0.019	0.029	0.658	0.511
	F3-novelty	0.322 ***	0.038	8.487	<0.001
	F4-cultural	0.288 ***	0.035	8.219	<0.001
	Ovl.Vis.Exp	0.191 ***	0.028	6.738	<0.001
	Ini.Att.Lat	0.050	0.032	1.553	0.121
	1st.Fix.Dep	−0.014	0.026	−0.551	0.582

Note: *** *p* < 0.001.

**Table 13:** Performance metrics of the Random Forest Regression model for predicting product attractiveness.

*R* ^2^	RMSE (Test)	MAE (Test)	OOB Error (MSE)	Validation MSE	Test MSE
0.340	0.788	0.657	0.667	0.611	0.621

**Table 14:** Random Forest feature importance metrics and rankings for predicting product attractiveness.

	Mean Decrease in Accuracy (MDA)	Rank (MDA)	Mean Decrease in Impurity (MDI)	Rank (MDI)
F3-novelty	0.271	1	65.352	1
F4-cultural	0.243	2	57.484	2
F1-aesthetic	0.101	3	38.796	6
F2-functional	0.094	4	39.367	5
Ovl.Vis.Exp.	0.087	5	51.494	3
Ini.Att.Lat.	0.040	6	44.949	4
1st.Fix.Dep.	−0.010	7	33.858	7

## Data Availability

The original contributions presented in this study are included in the article. Further inquiries can be directed to the corresponding author(s).
